# Ultrasound‐Assisted Synthesis and Comprehensive Characterization of Nanosized VO(II), Fe(III), and Ru(III) Complexes: From Density Functional Theory/Nonlinear Optical Properties to Pharmaceutical Applications and Docking Insights

**DOI:** 10.1002/open.70229

**Published:** 2026-05-22

**Authors:** R. A. El‐Kasaby, Eida S. Al‐Farraj, Senem Akkoc, Amal H. Alsehli, Maher Fathalla, Samir A. Abdel‐Latif, Mashael A. Alghamdi, Ahmed M. Abu‐Dief

**Affiliations:** ^1^ Department of Chemistry College of Science Taibah University Madinah Saudi Arabia; ^2^ Chemistry Department Women's College for Arts Science and Education Ain Shams University Cairo Egypt; ^3^ Department of Chemistry College of Science Imam Mohammad Ibn Saud Islamic University (IMSIU) Riyadh Saudi Arabia; ^4^ Department of Basic Pharmaceutical Sciences Faculty of Pharmacy Suleyman Demirel University Isparta Turkey; ^5^ Faculty of Engineering and Natural Sciences Bahcesehir University Istanbul Turkey; ^6^ Department of Chemistry Faculty of Science Islamic University of Madinah Madinah Saudi Arabia; ^7^ Department of Chemistry Faculty of Science Capital University (Formerly Helwan University) Cairo Egypt; ^8^ Department of Chemistry Faculty of Science Sohag University Sohag Egypt

**Keywords:** density functional theory calculations, isatin based imine ligand, molecular docking, nanosized metal complexes, pharmaceutical applications, ultrasound‐assisted synthesis

## Abstract

This research details the preparation of ultrafine particulate matter through high‐frequency sound wave‐facilitated chemical assembly. The ultrasonic irradiation method enhances reaction kinetics, facilitates uniform nanostructure formation, and provides an eco‐friendly approach to complex synthesis. Density functional theory calculations were employed to investigate the electronic structures, optimized geometries, and stability of the complexes. The theoretical findings provide insights into the molecular orbitals, charge distribution, and reactivity indices of the complexes. Furthermore, the pharmaceutical potential of the synthesized complexes was assessed through in vitro antimicrobial and anti‐cancer assays, revealing enhanced bioactivity compared to the free ligand. The antioxidant activity of nanosized complexes was assessed using the DPPH radical scavenging assays, highlighting their potential as effective free‐radical inhibitors. The results indicate the biological activity of the compounds following the sequence: CHBPIRu > CHBPIV > CHBPIFe > CHBPI ligand. Molecular docking simulations were performed to explore the binding interactions of the complexes with biological macromolecules, demonstrating strong affinity towards key enzymatic targets. The findings from this study highlight the synergistic effects of nanostructuring and ultrasound‐assisted synthesis in enhancing the physicochemical and biological properties of transition metal complexes. The combination of experimental and theoretical approaches provides a comprehensive understanding of these novel hydrazone‐based metal complexes.

## Graphical abstract

Ultrasound‐assisted synthesis enabled the formation of ultrafine hydrazone‐based metal complexes with controlled nanostructures and enhanced stability. Density functional theory revealed optimized geometries and electronic properties. The nanosized complexes exhibited superior antimicrobial, anticancer, and antioxidant activities, supported by molecular docking results, highlighting strong target binding and the synergistic impact of nano‐structuring on biological performance.

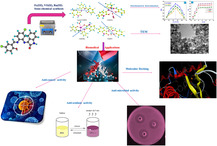

## 1 Introduction

The field of medicinal inorganic chemistry has gained significant momentum in recent decades, particularly with the development of transition metal complexes as potential therapeutic agents [1, 2, 3]. Nanotechnology has emerged as a pivotal field of research, impacting various scientific domains, including materials science, pharmaceuticals, and catalysis [4, 5, 6, 7]. Recent scientific focus has intensified on developing nanoscale complexes incorporating transition metals. These materials attract considerable interest owing to their distinctive structural configurations, unconventional physical and chemical behaviors, and promising utility in medical and biological contexts [8]. Among the vast array of transition metal complexes, vanadium (VO(II)), iron (Fe(III)), and ruthenium (Ru(III)) have demonstrated remarkable biological, catalytic, and electronic properties, making them suitable candidates for pharmaceutical and industrial applications [9, 10]. Moreover, vanadyl (VO(II)), iron (Fe(III)), and ruthenium (Ru(III)) complexes have emerged as promising candidates for various biomedical applications, including anticancer, antimicrobial, and antioxidant therapies. These metals are known not only for their rich coordination chemistry but also for their redox activity, biocompatibility, and relatively low toxicity when appropriately formulated [11, 12]. Coupling these metals with biologically active ligands such as isatin derivatives further enhances their therapeutic potential by allowing specific interactions with cellular targets, including DNA and enzymes. These ligands coordinate with metal centers to form stable and bioactive metal complexes with enhanced biological efficacy. Isatin (1*H*‐indole‐2,3‐dione) and its derivatives represent a privileged class of heterocyclic compounds widely explored for their pharmacological properties. Their scaffold can be readily modified to introduce a variety of functional groups, thus offering versatility in ligand design [13]. Isatin‐based compounds have demonstrated a broad spectrum of biological activities such as anticancer, antibacterial, antifungal, antiviral, anti‐inflammatory, and anticonvulsant effects [14]. The incorporation of isatin moieties into metal complexes has been shown to increase lipophilicity, facilitate cell membrane penetration, and enhance binding affinity to biological macromolecules. Moreover, the electron‐rich nature of isatin allows for strong coordination with transition metals, forming stable complexes with diverse geometries and bioactivities [15, 16]. In recent years, ultrasound‐assisted synthesis has emerged as an efficient and eco‐friendly method for preparing nanosized metal complexes. Ultrasonic irradiation facilitates rapid nucleation, enhances reaction kinetics, and promotes the formation of uniform nanostructures with controlled morphology [17]. Compared with conventional synthetic approaches, ultrasound‐assisted methods offer improved yields, reduced reaction times, and minimized by product formation, making them an attractive alternative for synthesizing nanoscale materials [18]. This is due to its ability to generate localized hot spots and high‐pressure conditions through acoustic cavitation. These benefits align well with the principles of green chemistry and support the scalable production of nanosized coordination compounds with desirable features for biomedical use. In parallel with experimental approaches, computational tools such as DFT calculations and molecular docking simulations have revolutionized in the field of coordination chemistry by providing deep insights into the structural, electronic, and pharmacological properties of metal complexes. DFT studies offer a quantum mechanical perspective on electronic structures, binding affinities, and reactivity indices of synthesized complexes, enabling the prediction of their stability and bioactivity [19]. Meanwhile, molecular docking studies serve as essential computational tools for assessing the interaction of metal complexes with biomolecular targets, shedding light on their potential as therapeutic agents [20]. Given the significant implications of transition metal complexes in medicinal and materials chemistry.

This study aims to synthesize and characterize nanosized like rods VO(II), Fe(III), and Ru(III) complexes using a novel 3‐{5‐chloro‐2‐[(5‐chloro‐2‐hydroxy‐benzylidene)‐amino]‐phenylimino}‐1,3‐dihydro‐indol‐2‐one ligand under ultrasound‐assisted conditions. The synthesized complexes thoroughly analyzed using various spectroscopic and structural techniques to elucidate their composition and morphology. Additionally, DFT calculations employed to study their electronic properties, while molecular docking simulations explores their pharmaceutical potential. The anticipated findings provided valuable insights into the structure–activity relationships of nanosized transition metal complexes and their prospective applications in drug discovery.

## 2 Experimental

### 2.1 Starting Chemicals and Reagents

Throughout this investigation, only chemicals and solvents of the highest quality, classified as reagent grade, were utilized. These substances were accepted and implemented directly from their original sources without undergoing any additional purification processes. The initial reagents comprised precursor compounds required for synthesizing Schiff base coordinating agents such as 8‐aminoquinoline, 5‐chloro‐2‐hydroxybenzaldehyde, ethanol, acetone, N,N′‐dimethylforamide (DMF), and N,N′‐dimethylsulfoxide (DMSO) products were used without distillation. In the process of crafting imine metal compounds, a selection of transition metal salts served as crucial reactants for the synthesis as follows: ruthenium(III) chloride hexahydrate (RuCl_3_·6H_2_O), vandadyl acetyl acetone (VO(acac)_2_), and iron(III) nitrate nonahydrate Fe(NO_3_)_3_·9H_2_O.

### 2.2 Instrumentation

The complete toolkit of measurement devices and experimental procedures utilized throughout this study appears in the supplemental documentation.

### 2.3 Synthesis of Tetra‐Dentate CHBPI Imine Ligand

**SCHEME 1:**
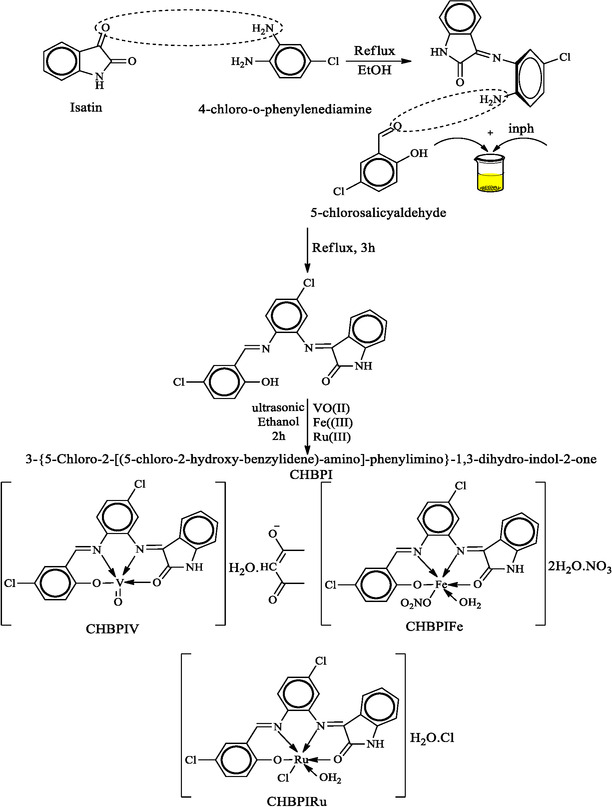
Synthetic pathway of the investigated CHBPI ligand and its complexes.

The synthesis of the 3‐{5‐chloro‐2‐[(5‐chloro‐2‐hydroxy‐benzylidene)‐amino]‐phenylimino}‐1,3‐dihydro‐indol‐2‐one (CHBPI) ligand involved a meticulous condensation process. Initially, 4‐chloro‐*o*‐phenylenediamine was measured at a concentration of 5 millimoles, which corresponds to 0.708 g, and dissolved in 25 milliliters of ethanol. This ethanolic solution was carefully combined with another 25 milliliters of ethanol in which 5 millimoles (0.735 g) of isatin had been previously dissolved. Following this, 5 millimoles (0.785 g) of 5‐chloro salicylaldehyde, also dissolved in 25 milliliters of ethanol, was gradually added to the mixture. The mixture underwent reflux for 3 h, producing a characteristic colored solid. This precipitate was isolated via vacuum filtration and cleansed with ethanol to remove solvent residues and impurities. The purified compound was then dried in a desiccator containing anhydrous calcium chloride to ensure complete moisture removal, yielding the pure CHBPI ligand. This synthetic pathway is illustrated in Scheme 1.

**CHBPI** ^1^H‐NMR (400 MHz, DMSO‐d_6_): d(ppm) 11.53 (s, 1H, OH), 8.89 (s, 1H, =CH), 8.05 (s, 1H, NH), 7.90 (s, 1H, H‐C6, *o*‐Cl), 7.88 (d, 2H, Arm), 7.56–6.65 (d, 3H, Arm, 5‐chlorosalicylaldehyde) 7.91 (s, 1H, H/C7, isatin), 6.81 ‐ 6.77 (t, 3H, H/C6, isatin). ^13^C‐NMR (δ, ppm) in DMSO‐*d*_6_: *164.27 ppm* (*C*_*q*_ *=O*), *160.11 ppm* (*CH = N*), *158.96* (*C*_*q*_ *=N*), *153.12* (*C*_*q*_ *‐OH*), 150.97, 146.52, 144.36, 142.21, 140.21, 133.16, 132.92, 130.48, 129.85, 125.38, 121.22, 119.84, 115.44, 111.16, 110,23, 107.59, and 103.29.

### 2.4 Sono‐Chemical Synthesis of Ru(III), VO(II), and Fe(III) Complexes With 3 {5‐Chloro‐2‐[(5‐Chloro‐2‐Hydroxy‐Benzylidene)‐Amino]‐Phenylimino}−1,3‐Dihydro‐Indol‐2‐One Ligand

Sono‐chemical preparation employs ultrasonic energy to accelerate chemical processes. This technique proves highly effective for producing metal complexes, such as Salen‐based structures, by significantly increasing reaction speed, boosting product yields, and enhancing purity. The synthesis procedure involves preparing a methanolic solution (20 mL) containing Fe(NO_3_)_3_ · 9H_2_O, VO(acac)_2_, or RuCl_3_ · 6H_2_O (2 mmol). This solution is gradually introduced into a separate methanolic solution of the ligand (2 mmol) while undergoing continuous 40 kHz ultrasonic irradiation. Sonication proceeds at ambient temperature for 1–2 h, facilitating complex formation (Scheme 1). The resulting precipitate is isolated via filtration, purified through sequential ethanol and ether washes, and finally desiccated under vacuum conditions.

### 2.5 Estimation of Stoichiometry of Complexes

The study utilized ligand exchange and Job's approach in solution‐based environments to evaluate the stability and stoichiometry of metal‐ligand complexes [21, 22, 23]. The absorbance of the system was recorded as both the metal ion and CHBPI ligand were incrementally added to the mixture. Sonication was applied for 10 min post‐combination to achieve a homogeneous distribution within the test samples. The obtained absorbance data was then graphically presented with respect to the mole fraction of the ligand in the mixture (expressed as [CHBPI] / [CHBPI] + [M]) and the molar ratio of ligand to metal ([CHBPI] / [M]). This technique provided insights into the interaction dynamics and the optimal stoichiometry for complex formation.

### 2.6 Evaluation of the Apparent Constant of Complexes

The derived formation constants (K_f_) for the complexes synthesized in solution were ascertained through spectrophotometric analysis, employing the continuous variation technique [24, 25]. The data was interpreted according to the formula designed.

For a 1:1 molar ratio:

(1) $$k_{f}=\frac{\frac{A}{A_{M}}}{C{\left(1-A/A_{m}\right)}^{2}}\;$$

The absorbance value *A*_M_ corresponds to the peak maximum, indicating the point of optimal complex formation. Meanwhile, a represents any absorbance measurement taken along the descending portion of the peak curve. The initial metal ion concentration is designated as C. For evaluating thermodynamic stability and calculating Gibbs free energy changes (ΔG*) within these chelate complexes, the subsequent equation was applied:

(2) $$\Delta G\;=\;‐RT\;\ln(K_{f})$$

The equilibrium constant *K*_f_ governs chelate complex formation, where its temperature dependence follows the relationship (2). In this expression, *R* denotes the ideal gas constant, while *T* signifies absolute temperature expressed in kelvins.

### 2.7 Measurements of Magnetic Susceptibility

Magnetic susceptibility measurements serve as a fundamental method for investigating the electronic structure of chemical compounds. The effective magnetic moment (μ_eff_) can be calculated from molar magnetic susceptibility ($\chi_{M}$) through the relationship [26, 27]:

(3) $$\mu_{\mathrm{eff}}=2.83\;\sqrt{\chi_{M}^{\prime }T}$$

where $\chi_{M}^{\prime }$ represents the corrected molar magnetic susceptibility, obtained by subtracting the diamagnetic correction from the observed molar susceptibility ($\chi_{M}$):

(4) $$\chi_{M}^{\prime }=\chi_{M}‐(\mathrm{diamag}.\mathrm{corr})$$

In these equations, *μ*_eff_ is expressed in Bohr Magnetons (*B*_M_), T denotes the temperature in Kelvin, and $\chi_{M}^{\prime }$ is the corrected parameter that accounts for both paramagnetic and diamagnetic contributions.

### 2.8 Spectrophotometric Studies

The detailed for spectrophotometric studies of the prepared CHBPI ligand and its complexes is shown in supporting information

### 2.9 Examining the Thermal Degradation and Reaction Kinetics

Employing thermogravimetric analysis (TGA) in conjunction with the Coats–Redfern relationship enables a thorough examination of the thermal degradation kinetics of the complexes, offering critical insights into their thermal stability, which is essential for their characterization and potential application assessment. This methodology delves into the intricacies of material behavior under varying thermal conditions, offering valuable insights into the physical and chemical transformations that occur. Given that thermal degradation is a kinetically governed process, the kinetic parameters of these complexes can be deduced using the Coats–Redfern equation, which is presented below [28, 29, 30]:

(5) $$\mathrm{Log}\;\left[\frac{\mathrm{Log}\;\left(\frac{W_{\infty }}{W_{\infty }‐w}\right)}{T^{2}}\right]=\mathrm{Log}\;\left[\frac{AR}{\emptyset E^{*}}\left(1‐\frac{2RT}{\emptyset E^{*}}\right)\right]‐\frac{E^{*}}{2.303RT}$$

In the aforementioned equation, (E*) symbolizes the activation energy, (*R*) is the universal gas constant, (*T*) is the temperature, and (*W*) and (*W*_∞_) represent the residual and initial mass of the complex, respectively. By assuming (1‐2RT/E*) to be approximately equal to 1, the equation simplifies, allowing for the computation of the kinetic parameters.

The enthalpy of degradation (ΔH*), entropy (ΔS*), and activation energy change in free energy (ΔG*) can be derived from the following expressions:

(6) $$\Delta H^{*}\;=\;E^{*}\;‐\;RT$$

(7) $$\Delta S^{*}\;=\;2.303R\;\log\frac{Ah}{K_{B}T}$$

(8) $$\Delta G^{*}\;=\;\Delta H^{*}\;‐\;T\Delta S$$

Here, (h) represents Planck's constant, (*R*) is the Boltzmann's constant, (*A*) is the pre‐exponential factor, and (*T*) signifies the absolute temperature.

### 2.10 DFT and Docking Studies

DFT and docking analyses are two interconnected computational methods commonly employed in the realm of molecular modeling and drug design to predict the interaction between molecules and understand their structural properties at the quantum mechanical level. These studies aim to elucidate how a small molecule, known as a ligand, binds to a larger molecule, typically a protein, to form a stable complex. DFT is used to compute the electronic structure and energy of molecules, which is fundamental for comprehending the chemical reactivity and bonding patterns. It serves as a theoretical framework that allows researchers to simulate molecular interactions with a high degree of accuracy, while docking focuses on the spatial orientation of the ligand within the binding site of the target molecule to predict the most favorable binding configuration and affinity. Combining these two approaches enhances the predictive power in the quest to identify potential drug candidates and optimize their molecular structures for better therapeutic efficacy.

#### 2.10.1 Computational Details

The computational analysis of the molecular structures in this study was performed with the Gaussian‐09W software, which was employed for the purpose of energy minimization. A single crystal X‐ray diffraction method was not utilized to elucidate the detailed geometric characteristics of the compounds [31]. Instead, DFT approach was applied to optimize the geometrical conformations of the synthesized compounds at the B3LYP functional level [32, 33, 34, 35, 36]. For the free CHBPI ligand, the optimization was conducted at the B3LYP/6‐311G (d, p) level, while for the chelate structures of the metal ions Fe, Ru, and VO, the basis set of 6‐311G (d, p)‐LANL2DZ was utilized [37, 38]. The use of the 6‐311G(d, p) basis set for the ligand ensures accurate description of light atoms, while a mixed basis set approach involving effective core potentials for the metal centers was adopted to account for relativistic effects and reduce computational cost, providing a balanced and reliable treatment of the metal–ligand system at the B3LYP functional level.

The optimization process involved releasing all bond angles, dihedral angles, and bond lengths to achieve the lowest energy state. The quantum chemical properties of the ligand and its metal complexes were further investigated using the DFT framework [39, 40]. This theoretical method allowed for a comprehensive examination of various aspects, including energy optimization, geometric parameters, and the construction of molecular orbital (MO) diagrams. The Gauss View 5 [41] software served as a tool to visualize these MO configurations, which are essential for understanding the electronic properties of the compounds [42]. Additionally, the nonlinear optical properties, such as the polarizability anisotropy (Δα), dipole moment (μ), and the average polarizabilities along the x, y, and *z* axes (<β> and <α>), were computed from the Cartesian coordinates of the optimized structures.

#### 2.10.2 Molecular Docking Approaches

The MOE2022 software package was utilized to conduct molecular docking studies [43]. Protein structures with PDB identifiers 3HB5, 3QLW, and 3IF5, corresponding to breast cancer, *Candida albicans*, and *Micrococcus luteus*, respectively, were retrieved from the Protein Data Bank (PDB). Throughout the docking simulations, which were performed under default conditions, the selection of optimal conformations was guided by several criteria, including computed binding scores and the E conformations values. The reliability of the molecular docking protocol was validated through Root Mean Square Deviation (RMSD) analysis by redocking the co‐crystallized ligand into the active site of the target protein. The obtained RMSD values were found to be within the acceptable threshold (≤2.0 Å), confirming the accuracy and reproducibility of the docking method in predicting the correct binding pose. The low RMSD values indicate a strong agreement between the experimental crystallographic conformation and the docked conformation, thereby validating the robustness of the docking results and supporting the credibility of the predicted ligand–protein interactions

### 2.11 Biological Assessment Approach

#### 2.11.1 Anti‐Pathogenic Activity

The biological screening of the compounds under study was performed in vitro against both gram‐negative bacteria, namely *Escherichia coli* and *Serratia marcescens*, as well as gram‐positive bacteria, *Micrococcus luteus*. The antifungal properties were evaluated using potato Dextrose Agar to combat species such as *Getrichm candidum*, *Aspergillus flavus*, and *Fusarium oxysporum*. The compounds were initially dissolved in DMF to create stock solutions. The typical methodology involved creating wells in the inoculated nutrient agar plates using a borer and filling these with varying concentrations of the test solution via a micropipette. These plates were then maintained at 37°C for 24 h for bacterial cultures and 48 h for fungal cultures. Ofloxacin and fluconazole used as standard comparatives for the bacterial and fungal assays, respectively. The antimicrobial impact was discernible through the emergence of distinct inhibition zones around the wells, which were measured post‐incubation in millimeters [44]. In the preparation of complexes, sterile petri dishes were filled with 20 ml of Muller Hinton Agar for bacterial studies and 20 ml of potato Dextrose Agar for yeast examinations.

After allowing the inoculums to dry for 10 min on the agar surface, the discs containing the complexes at 15 and 25 mg/ml concentrations were aseptically placed on the plates [45]. This was followed by a 30‐min diffusion period at room temperature. Positive controls included ofloxacin for bacterial cultures and fluconazole for fungal assays. Subsequent incubation occurred at 37°C for 24 h for bacteria and within the range of 28–35°C for 24 h for fungi. The sensitivity of the microbes to the compounds was recorded by measuring the zones of growth inhibition on the agar around the discs, also in millimeters, after the specified incubation periods.

#### 2.11.2 Assessing Tumor‐Suppressing Potential: A Ligand and Its Metallic Derivatives Under Scrutiny

The anticancer properties of CHBPIV, CHBPIFe, and CHBPIRu complexes were evaluated against three carcinoma cell lines: Hep‐G2 (liver cancer), MCF‐7 (breast cancer), and HCT‐116 (colon cancer). Cytotoxicity screening was performed in vitro using the sulforhodamine B (SRB) assay methodology, a validated approach for quantifying cellular growth inhibition. Cancer cells were seeded in 96‐well plates at 10,000 cells per well and incubated for 24 h. This pre‐incubation period ensured optimal cell attachment prior to compound exposure. Metal‐based complexes were dissolved in DMSO to generate concentration gradients (0, 1.65, 3.125, 6.25, 12.5, 25, and 50 mM), leveraging DMSO's solvation properties for cellular delivery. After compound administration, plates underwent 48‐h incubation at 37°C to assess therapeutic effects. Post‐incubation, cells were fixed and washed to remove residual compounds. Viable cells were quantified via Sulforhodamine B staining, with acetic acid and Tris‐EDTA buffer employed to remove unbound dye from adherent cells. This methodology enabled precise determination of IC_50_ values the concentration yielding 50% proliferation inhibition—calculated using standardized procedures referenced in the literature [46, 47, 48].

(9) Inhibition concentration (IC) % = (Control O.D ‐ Ligand O.D) × 100 / Control O.D

#### 2.11.3 Antioxidant Activity

The evaluation of the antioxidant capabilities of the prepared compounds was performed using the DPPH free radical scavenging method as in literature [49, 50]. The detailed method is shown in supporting in formation,

All biological experiments were performed in triplicate, and the results are expressed as mean ± standard deviation (SD). The statistical analysis of the antimicrobial activity data, including the calculation of standard deviation (SD), was carried out using Microsoft Excel.

## 3 Results and Discussion

### 3.1 The Comprehensive Analysis and Depiction of the CHBPI Ligand and Its Associated Complexes

Experimental evidence from stoichiometric analysis, elemental composition (CHN) data, and conductivity assessments consistently verifies that ligand CHBPI forms 1:1 molar ratio complex with vanadium, iron, and ruthenium ions. CHN analytical results align with predicted molecular frameworks, corroborating the structural integrity of these complexes [27, 32, 33, 34].

### 3.2 FTIR Spectrum

IR spectroscopy is a vital tool for identifying functional groups and confirming coordination modes in ligands and their metal complexes. It provides insight into bond vibrations and shifts that reveal metal–ligand interactions and structural modifications upon complexation. The CHBPI ligand and its metal complexes exhibit characteristic infrared absorption bands summarized in Table 1. Diagnostic vibrations for OH, carbonyl (C=O), and imine (CH—N) groups reveal structural features and coordination behavior. Notably, the ligand's CH—N vibration appears at 1605 and 1624 cm^−1^. Upon complexation, this band shifts downward: CHBPIV complexes show absorptions at 1602 and 1571 cm^−1^, CHBPIFe at 1605 and 1575 cm^−1^, and CHBPIRu at 1614 and 1598 cm^−1^. This hypsochromic shift confirms azomethine nitrogen participation in metal binding [51, 52]. A sharp peak at 3465 cm^−1^ in the free ligand spectrum corresponds to hydroxyl stretching vibrations. Following complex formation, a broad absorption emerges between 3447–3463 cm^−1^, indicative of hydrated water molecules. Elemental analysis data in Table 1 corroborates this hydration state. The free ligand exhibits a C—O absorption band at 1295 cm^−1^. Upon complex formation, this peak shifts to lower frequencies: 1267 cm^−1^ for CHBPIV, 1271 cm^−1^ for CHBPIFe, and 1261 cm^−1^ for CHBPIRu. This downward shift indicates hydroxy group involvement in C‐O‐M bonding following deprotonation [53, 54]. Similarly, the ligand's carbonyl stretching vibration at 1734 cm^−1^ undergoes a reduction in wavenumber upon coordination, appearing at 1687 cm^−1^ (CHBPIV), 1692 cm^−1^ (CHBPIFe), and 1690 cm^−1^ (CHBPIRu). These changes confirm oxygen atom coordination from the C=O group to the metal center. New vibrational bands emerge in the complexes’ FT‐IR spectra, Metal‐oxygen (M‐O) stretches appear at 442 cm^−1^ (CHBPIV), 455 cm^−1^ (CHBPIFe), and 440 cm^−1^ (CHBPIRu). Distinct absorptions between 535 and 567 cm^−1^ correspond to characteristic M‐N stretching vibrations [27, 32, 33, 34, 55]. These spectral modifications collectively demonstrate metal‐ligand coordination through both oxygen and nitrogen atoms.

**TABLE 1:** Evaluating the quantitative and physicochemical attributes of CHBPI Schiff base ligand and its associated compound formations.

Compounds	**Empirical formula** **(formula weight)**	Color	**(M. p.) and Decomp. Temp., ** ^**o**^ **C**	**Λ**_**m**_ **, ** **Ω** ^**−1**^ **cm** ^**2**^ **mol** ^**−1**^	**µ**_**eff**_ **, ** **B.M.**	**Analysis, %** **Found, Calc.**			IR, cm^−1^					
						C	H	N	υ OH/H_2_O	(CH=N)υ_ph_	υC=O	υC‐O	υ M‐N	υC‐O
**CHBPI**	C_21_H_13_Cl_2_N_3_O_2_ (410.25)	yellow	212	—	**—**	61.53 (61.48)	3.22 (3.19)	10.18 (10.24)	3465	1624 1605	1734	1295	—	—
**CHBPIV**	C_26_H_21_Cl_2_N_3_O_6_V (593.13)	orange	>300	59.40	1.78	52.67 (52.63)	3.54 (3.57)	7.12 (7.08)	3447	1602 1571	1687	1267	535	442
**CHBPIFe**	C_21_H_18_Cl_2_N_5_O_11_Fe (643.14)	Blue	>300	62.17	5.34	39.36 (39.22)	2.93 (2.82)	10.97 (10.89)	3463	1605 1575	1692	1271	567	455
**CHBPIRu**	C_21_H_16_Cl_4_N_3_O_4_Ru (617.25)	Violet	>300	60.56	1.93	40.80 (40.86)	2.65 (2.61)	6.85 (6.81)	3451	1614 1598	1690	1261	560	440

### 3.3 ^1^H‐NMR and ^13^C‐NMR Spectral Evaluations

**FIGURE 1:**
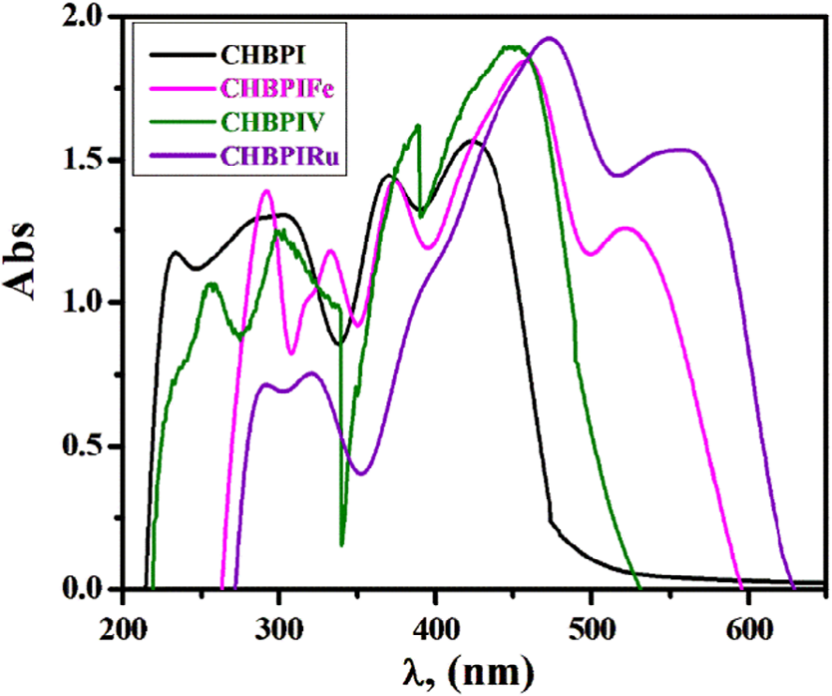
Electronic absorption spectra of CHBPI ligand and its complexes in DMF at 298 K.

**FIGURE 2:**
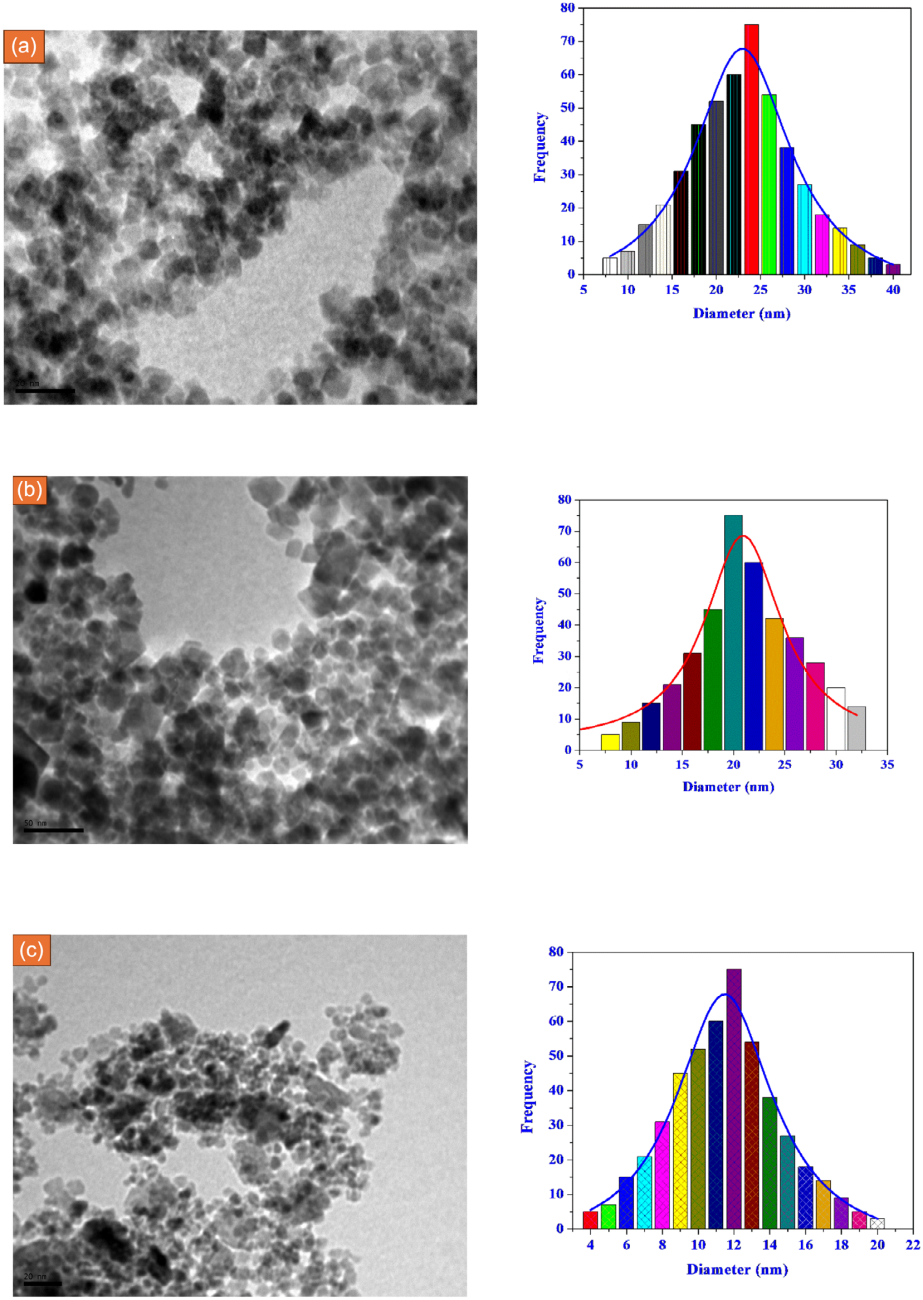
Visualizing nanoscale structures and particle size distribution analysis of synthesized nanoparticles (a) CHBPIFe, (b) CHBPIV, and (c) CHBPIRu metal chelates.

The CHBPI ligand was thoroughly examined using ^1^H NMR spectroscopy, where DMSO‐d_6_ served as the solvent and TMS functioned as the internal reference. The recorded spectrum, illustrated in Figure 1S, displays a singlet at 11.53 ppm, attributed to the phenolic –OH proton. Another distinct singlet appearing at 8.89 ppm corresponds to the proton attached to the imine (CH=N) group [27]. In addition, a singlet at 8.05 ppm confirms the presence of the –NH proton. Within the aromatic region, the spectrum demonstrates multiple splitting patterns, including doublet and triplet signals. These were observed at 7.90 ppm (s, 1H, H–C6, o‐Cl), 7.88 ppm (d, 2H, aromatic protons), and within the range of 7.56–6.65 ppm (d, 3H, aromatic protons from 5‐chlorosalicylaldehyde). Furthermore, additional resonances were detected at 7.91 ppm (s, 1H, H–C7, isatin) and 6.81–6.77 ppm (t, 3H, aromatic protons of the isatin ring), confirming the expected proton environment of the ligand structure [15]. Moving to the ^13^C‐NMR analysis, the data obtained in a DMSO environment is presented in Figure 2S. These data offer further insight into the ligand's structure, highlighting chemical shifts at 164.27 ppm for the carbon atom in the C=O bond, 160.11 ppm for the carbon bonded to the CH=N group, and 158.96 ppm for the carbon atom in the C=N bond. Other significant shifts were identified at 153.12 ppm for the C–OH bond, as well as various additional shifts at 150.97, 146.52, 144.36, 142.21, 140.21, 133.16, 132.92, 130.48, 129.85, 125.38, 121.22, 119.84, 115.44, 111.16, 110,23, 107.59, and 103.29 ppm. These distinct chemical shifts serve as markers for the various carbon atoms that constitute the intricate structure of the ligand. The combination of these spectroscopic techniques and their findings confirm the anticipated structural features of CHBPI, thereby reinforcing its characterization.

### 3.4 Examination of the Fundamental Constituents and Their Respective Contributions to the Molar Conductivity of Substances

The presented data for the CHBPI ligand and its corresponding complexes with different metal centers reveals that the ligand exhibits tetradentate behavior, as deduced from the microanalytical outcomes. This implies that the ligand coordinates with the metal ions through four binding sites, which is consistent with the formation of 1:1 metal‐to‐ligand complexes. The proposed molecular formulas for the complexes are C_26_H_21_Cl_2_N_3_O_6_V, C_21_H_18_Cl_2_N_5_O_11_Fe and C_21_H_16_Cl_4_N_3_O_4_Ru, reflecting the composition of the vanadium, iron, and ruthenium complexes, respectively (Table 1). The molar conductivity measurements of 10^−3^ M solutions of these complexes in ethanol, presented in the same table, are 59.40, 62.17, and 60.56 Ω^−1^ cm^2^ mol^−1^. These values suggest that the complexes are ionic in character, which supports the hypothesis that they are electrolytic species [27, 32, 33, 34, 56]. This conclusion is drawn based on the observed trends in conductivity, a key property that distinguishes between molecular and ionic compounds in solution. In the context of these findings, the CHBPI ligand's ability to create stable and soluble complexes with varying metal ions is notable and may have implications for further studies in coordination chemistry and potential applications.

### 3.5 Electronic Absorption Spectra

The absorption spectra of DMF‐dissolved ligand and its corresponding metal complexes were recorded over a 200–800 nm wavelength span at a temperature of 298 K. The data, including absorption regions and molar extinction coefficients, are presented in Table S1 and Figure 1. The ligand is characterized by absorption bands at 232, 295, 368, and 422 nm, which can be ascribed to transitions such as π → π*, n → π*, and intra‐ligand events arising from the CH=N, C=N, and C=O groups, contributing to its natural yellow hue [15, 57, 58]. Upon interaction with metal ions, substantial spectral variations are observed, revealing alterations in the ligand's molecular framework and the metal's electronic environment. This suggests complex formation. For instance, the CHBPIV complex presents distinct bands at 357, 300, 386, and 448 nm, which are associated with π → π*, n → π*, and ligand‐to‐metal charge transfer (LMCT) transitions, along with d→d transitions. In contrast, the CHBPIFe complex displays bands at 291 nm for π → π*, 318 and 332 nm for n → π*, 372 and 459 nm for LMCT, and 522 nm for the d → d transition [59]. The CHBPIRu complex, on the other hand, shows bands at 291 nm (π → π*), 319 nm (n → π*), 399 and 473 nm (LMCT), and 553 nm (d → d transition) [60].

### 3.6 Magnetic Moment

Paramagnetic substances are characterized by their attraction to magnetic fields, whereas diamagnetic compounds experience repulsion in such environments. The magnetic susceptibility of these materials is indicative of their spatial orientation within the magnetic field, which in turn is influenced by the structure of their complexes. According to the established principle, μ_eff_ = [4S(S + l)]^0.5^, the magnetic susceptibility can be used to deduce the geometric arrangement of complexes. In the examined research, the magnetic susceptibilities of the compounds align closely with the theoretical framework provided by the equation. Specifically, the CHBPIRu chelate presents a magnetic moment of 1.93 Bohr Magnetons (B.M.), which is a significant indicator of its high‐spin octahedral geometry (Table 1). This empirical value is notably in line with the theoretical predictions for ruthenium (III) complexes, which typically exhibit high‐spin behavior [21]. Furthermore, the CHBPIFe complex demonstrates a paramagnetic nature with a magnetic moment of 5.34 B.M. This empirical finding corresponds well with the octahedral geometry expected for high‐spin iron (III) complexes, as suggested by the octahedral arrangement of the ligands around the central iron atom [9]. In contrast, the CHBPIV chelate presents a magnetic moment of 1.78 B.M., which aligns well with the theoretical predictions for a square pyramidal geometry in the context of a vanadium (IV) ion. This observation implies the presence of a high‐spin configuration, featuring four unpaired electrons, a property that is consonant with the typical electronic arrangement expected for such a structural motif in this category of complexes [61].

### 3.7 Microscopic Exploration: Imaging Fe(III), VO(II), and Ru(III) Nanoscale Complexes via Transmission Electron Microscopy

Transmission electron microscopy (TEM) analysis was carried out using an instrument operated at an accelerating voltage of 200 kV. Prior to imaging, the synthesized complexes were suspended in ethanol and subjected to ultrasonic treatment for 15 min to obtain a well‐dispersed and homogeneous solution. A drop of the resulting suspension was carefully placed onto a carbon‐coated copper grid and left to dry naturally at ambient conditions before examination.

The TEM images revealed that the Fe(III), VO(II), and Ru(III) complexes were predominantly present as uniformly distributed nanoparticles with clearly defined shapes and relatively narrow size distributions. Particle size analysis was conducted using ImageJ software (Figure 2) [62, 63, 64], from which the average particle diameters were calculated and are summarized below:

• **CHBPIV** Complex: Average particle diameter of approximately 23 nm.
• **CHBPIFe** Complex: Fe(III)‐based nanoparticles exhibited an average diameter of 21 nm.
• **CHBPIRu** Complex: Ru(III) nanoparticles showed an average diameter of 11 nm.

### 3.8 Thermal Analysis

Thermal analysis served to assess the robustness of the metal complexes and determine the presence of water molecules either within or outside the central metal ion's coordination environment [65, 66]. The thermograms revealed that the CHBPIV compound contained a single water molecule in a hydrated state, while the CHBPIFfe complex had two hydrated and a single coordinated water molecule. In the case of CHBPIRu, one water molecule was found to be hydrated and another coordinated. The thermal profiles of these complexes exhibited an initial stage of water loss, which involved the removal of either hydrated or coordinated water molecules. This was followed by the decomposition of the CHBPI ligand in the subsequent step, as presented in Table 2. The TGA analysis of CHBPIV exhibits five distinct degradation phases spanning temperatures from 38 to 590°C. Initially, between 38°C and 122°C, the material experiences a loss of one molecule of hydrated water, equating to a mass reduction of approximately 2.96%, closely matching the theoretical prediction of 3.03%. Subsequently, the second phase occurs at 125°C–200°C, characterized by the elimination of a portion of the ligand (C_5_H_7_O_2_), which results in a mass loss of 16.3%, slightly less than the calculated value of 16.6%. Moving to the third phase, which takes place at 210°C–320°C, there is the removal of another part of CHBPIV (C_6_H_3_ClO), with a mass loss of 21.3%, very close to the expected 21.4%. The fourth stage, observed at 320°C–445°C, involves the removal of a further ligand segment (C_7_H_4_ClN_2_), with a mass loss of about 26%, aligning well with the calculated 25.6%. Finally, the fifth phase between 450 and 580°C corresponds to the extraction of additional ligand components (C_8_H_5_NO), leading to a mass decrease of approximately 21.90%, which is almost the same as the predicted 22.09%. Throughout these thermal breakdowns, vanadium oxide (VO) remains as the residual product. The thermogravimetric analysis (TGA) of the CHBPIFe compound revealed a sequence of weight loss events that offered substantial insight into its molecular composition. At the onset, from 52°Cto 184°C, there was a notable mass reduction of 17.74%, which can be ascribed to the desorption of a molecule of water (·3H_2_O) along with a nitrate group (NO_3_). Subsequently, a second weight loss phase, amounting to approximately 27.2%, occurred in the temperature range of 185°C–282°C, corresponding to the removal of a C_6_H_3_ClNO_3_ functional group. Further thermal degradation of the CHBPIFe complex took place in two stages: from 285 to 400°C, the C_5_H_3_ClNO moiety was expelled, resulting in a mass loss of about 20.2%, and from 400°C to 510°C, a C_7_H_4_N segment was released, causing an additional decrease of 16.1% in mass. The final phase of the complex's thermal decomposition was observed between 520°C and 662°C, where a C_3_H_2_N portion was eliminated, contributing to a mass reduction of approximately 7.92%. The remnant material that withstood the thermal treatment was unmistakably identified as the metal oxide, FeO. The thermal analysis of the CHBPIRu chelate revealed a multistep degradation process. Initially, a loss of 11.5% (theoretical: 11.6%) in weight was discerned from 34°C to 149°C, which is indicative of the evaporation of two water molecules and a chlorine atom. Following this, a substantial weight reduction of 23.42% (theoretical: 23.5%) occurred in the temperature interval of 155°C–270°C, corresponding to the elimination of a C_6_H_3_Cl_2_ molecule. A subsequent degradation step, spanning from 280°C to 365°C, presented a mass loss of 20.48% (theoretical: 20.25%), which can be attributed to the release of the C_6_H_3_ClN organic group. The fourth stage of degradation, between 370°C and 490°C, exhibited a 14.96% (calculated: 15.07%) mass loss, which is associated with the removal of a C_5_H_3_ON molecule. In the fifth and final major stage, from 500°C to 610°C, a decrease of 10.50% (theoretical: 10.54%) in weight was noted, corresponding to the loss of the C_4_H_3_N species. Beyond 620°C, the degradation proceeded at a more gradual pace, culminating in the formation of RuO, with a total weight loss of 19.10% (calculated: 18.96%).

**TABLE 2:** Thermal decomposition steps, mass loss (%), final residue and thermokinetic activation parameters.

Complexes	Temp (^o^C)	Fragment loss, %		Weight loss, %	
		Molecular formula	M. Wt.	Found	Calc
CHBPIV	38–122	H_2_O	18	2.96	3.03
	125–200	C_5_H_7_O_2_	99	16.3	16.6
	210–320	C_6_H_3_ClO	127	21.3	21.4
	320–445	C_7_H_4_ClN_2_	152	26	25.6
	450–580	C_8_H_5_NO	131	21.90	22.09
Residue	>590	VO	67	11.5	11.2
CHBPIFe	52–184	·3H_2_O + NO_3_	116	17.74	18
	185–282	C_6_H_3_ClNO_3_	173	27.2	26.9
	285–400	C5H3ClNO	129	20.2	20.06
	400–510	C_7_H_4_N	102	16.1	15.8
	520–662	C_3_H_2_N	52	7.92	8.08
Residue	>670	FeO	72	10.80	11.1
CHBPIRu	34–149	·2H_2_O + Cl	72	11.5	11.6
	155–270	C_6_H_3_Cl_2_	145	23.42	23.5
	280–365	C_6_H_3_ClN	125	20.48	20.25
	370–490	C_5_H_3_ON	93	14.96	15.07
	500–610	C_4_H_3_N	65	10.50	10.54
Residue	>620	RuO	117	19.10	18.96

#### 3.8.1 Kinetic Parameter

The thermal degradation behavior of the synthesized complexes was examined using the Coats–Redfern kinetic model, which enabled the determination of key kinetic and thermodynamic parameters (Table 3). The analysis revealed that the metal complexes exhibit higher thermal stability compared with their corresponding activation energies (E*). As the degradation process progresses, a gradual increase in activation energy are observed, suggesting that the decomposition of the complexes proceeds at a slower rate than that of the free ligand. This observation confirms the enhanced thermal robustness of the metal chelates. In addition, the *negative entropy change* (Δ*S*)* values indicate a transition toward more ordered and compact structures during complex formation relative to the free ligands. The negative ΔS* coupled with *positive Gibbs free energy* (Δ*G*)* values further suggests that the decomposition reactions are non‐spontaneous under the applied conditions. Meanwhile, the *positive enthalpy values* (Δ*H*)* confirm that the thermal degradation of the complexes is an endothermic process. This endothermic behavior is further supported by the linear correlation obtained from the Arrhenius plots, which demonstrate consistent relationships among the calculated kinetic parameters throughout the decomposition stages. Overall, the results indicate that the thermal stability and degradation kinetics of the investigated complexes are closely interrelated, highlighting their significant resistance to thermal decomposition [67, 68, 69].

**TABLE 3:** Decomposition phases of analyzed compounds: thermokinetic initiation characteristics.

Complexes	E*, KJmol^−1^	A, S^−1^	ΔH*, KJmol^−1^	ΔG*, KJmol^−1^	ΔS*, Jmol^−1^K^−1^
**CHBPIV**	36	0.01	35.37	57.15	−272.30
			34.69	79.75	−278.17
			33.83	108.63	−282.26
			32.86	141.84	−285.30
			31.75	179.96	−287.79
**CHBPIFe**	43	0.006	42.64	75.66	−279.78
			41.69	108.20	−285.44
			40.78	139.50	−288.63
			39.84	172.25	−291.00
			38.71	211.98	−293.18
**CHBPIRu**	74	0.004	73.69	99.55	−281.08
			72.70	133.76	−288.02
			71.78	165.65	−291.50
			70.88	197.26	−293.91
			69.84	234.14	−296.03

### 3.9 Stoichiometry of Complexes in Solutions

**FIGURE 3:**
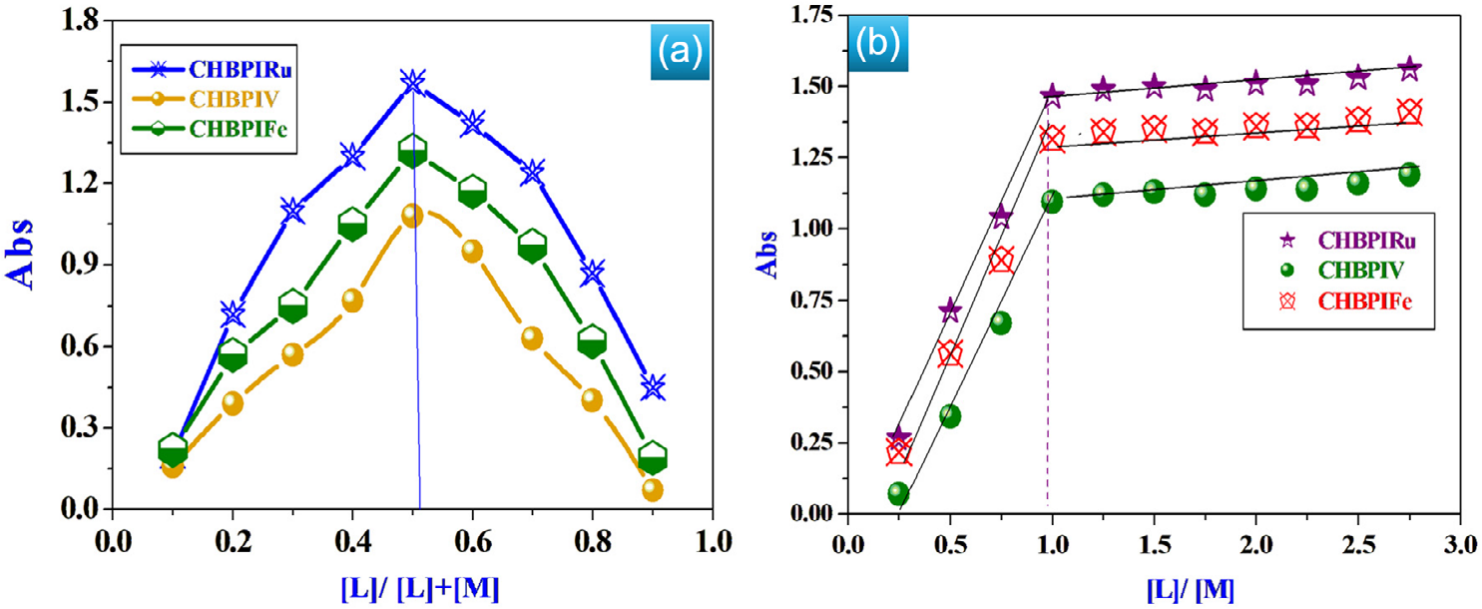
An illustrative depiction of continuous variation (a) and molar ratio (b) diagrams showcasing the behavior of CHBPI Schiff base complexes immersed in DMF at a concentration of 10^−4^ M under standard room temperature conditions (298 K).

A spectrophotometric analysis was conducted to investigate the equilibrium behavior of the synthesized complexes in solution using two standard approaches: the molar ratio method [15, 27, 32, 33, 34] and the continuous variation (Job's) method [70], as illustrated in Figure 3. The results obtained from these techniques confirmed the formation of a 1:1 metal‐to–ligand complex, as represented in Scheme 1. In the continuous variation method, the absorbance profile displayed a maximum at a ligand mole fraction of 0.51, indicating the formation of complexes with a stoichiometric ratio of one metal ion to one ligand molecule (Figure 3a). Similarly, the data derived from the molar ratio method supported this finding, revealing that the metal and ligand interact in a 1:1 ratio (Figure 3b). These consistent results from both methods strongly validate the proposed stoichiometric composition of the synthesized complexes.

### 3.10 The Formation Constants of the Investigated Complexes

The equilibrium formation constants (K_f_) of the CHBPI metal complexes were determined from spectrophotometric measurements using the continuous variation (Job's) method, following the procedures described in references [3, 9, 70]. As summarized in Table 4, the obtained K_f_ values reflect the high thermodynamic stability of the synthesized complexes in solution. The order of stability among the investigated complexes was found to increase as follows: CHBPIV < CHBPIFe < CHBPIRu, indicating that the ruthenium complex possesses the greatest structural stability within the series. Moreover, Table 4 also includes the corresponding stability constant (pK) and *Gibbs free energy change* (Δ*G*)* values, which provide further insight into the energetic and interaction characteristics of the metal–ligand systems. These parameters collectively confirm the strong binding affinity and enhanced stability of the CHBPIRu complex compared to the others.

**TABLE 4:** A Compilation of synthesized complex characteristics: formation constants (Kf), stability constants (pK), and Gibbs free energy changes (ΔG*) at 298 K temperature.

Complexes	Type of complex	***K***_**f**_	**Log *K***_**f**_	Δ*G* ^*^ ***, *** **KJmol** ^**−1**^
CHBPIV	1:1	3.65 × 10^4^	4.56	−26.02
CHBPIFe	1:1	4.25 × 10^4^	4.63	−26.40
CHBPIRu	1:1	6.15 × 10^4^	4.78	−27.32

### 3.11 Exploring pH Profile of the Complexes Under Inspection

**FIGURE 4:**
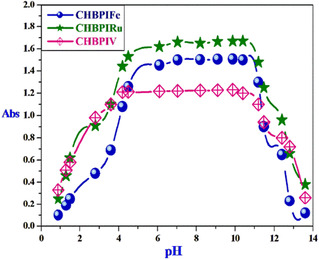
Graphic representation of the pH‐dependent behavior of CHBPI‐metal compounds in aqueous‐ethanol solutions.

The pH‐dependent absorption spectrum depicted in Figure 4 exhibits characteristic dissociation patterns, revealing a broad stability range of 4–11 for the investigated complexes. This extensive pH window suggests that the formation of the complex significantly enhances the stability of the associated CHBPI ligand. Consequently, these complexes are well‐suited for various biological applications, given that they remain intact across diverse physiological pH conditions [71].

### 3.12 DFT Insight

#### 3.12.1 Molecular Orbital Treatment

**FIGURE 5:**
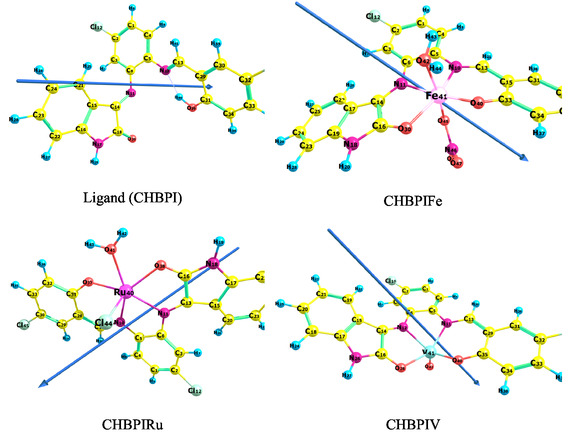
Optimized geometry, numbering system, and vector of dipole moment for the studied CHBPI ligand and its Fe, Ru, and VO chelates using B3LYP/6‐311G (d, p) and B3LYP/6‐311G (d, p)‐LANL2DZ level.

The ligand's geometry underwent optimization using density functional theory at the B3LYP/6‐311G (d, p) level to attain maximum structural stability (Figure 5). Analysis of dihedral angles in Table S2 reveals significant deviations from both 0 and 180°, confirming the ligand's non‐planar conformation. This structural feature enhances its capacity for chelate formation. Natural charge distribution data (Table 5) identifies potential coordination sites on the CHBPI ligand, with atoms N10 (−0.523), N11 (−0.375), O20 (−0.537), and O39 (−0.659) exhibiting substantial negative charges. These electrostatic characteristics indicate these atoms possess high suitability as coordination centers for Fe, Ru, and VO^2+^ metal ions.

**TABLE 5:** NBO charges calculated for the CHBPI ligand and its Fe, Ru, and VO chelates using B3LYP/6‐311G (d, p) and B3LYP/6‐311G (d, p) ‐LANL2DZ level.

	Ligand CHBPI	CHBPIFe	CHBPIRu	CHBPIV
N10	−0.523	−0.424	−0.420	−0.468
N11	−0.375	−0.351	−0.401	−0.478
O20	−0.537	−0.512	−0.608	−0.562
O39	−0.659	−0.595	−0.600	−0.607
M	—	0.430	0.552	0.750

#### 3.12.2 Geometry of Solid Chelates

Ground‐state geometries of iron, ruthenium, and vanadium chelates embedded within the CHBPI solid matrix (Figure 5) underwent optimization using mixed basis sets: B3LYP/6‐311G(d, p) for light atoms and LANL2DZ for metals. This computational refinement reveals structural characteristics of these metal‐organic systems. Table S2 details critical geometric parameters including dihedral angles, bond distances, and angular relationships for the coordinated complexes. Analysis indicates elongation in C5‐N10 and C18‐O20 bond lengths compared to the unbound ligand, consistent with coordinate bond formation involving metal cations and donor atoms (N10, O20). Conversely, shortened C6‐N11 and C31‐O39 distances suggest additional chelation sites at N11 and O39. Coordinate covalent bonding in metal‐nitrogen complexes typically yields longer M‐N bonds than ionic interactions, diminishing ionic character [72]. For Fe‐, Ru‐, and CHBPIV chelates, coordination occurs specifically at ligand atoms N10, N11, O20, and O39. Distorted octahedral geometry is evidenced by bond angles such as ∠C5‐N10‐Fe41 (113.122°), ∠O30‐Fe41‐O40 (98.849°), and ∠C13‐N10‐Fe41 (122.194°) [15, 27, 32, 33, 34]. Stability calculations confirm this distorted octahedral configuration as energetically favorable across all metal‐ligand systems studied.

#### 3.12.3 Global Reactivity Descriptors

**FIGURE 6:**
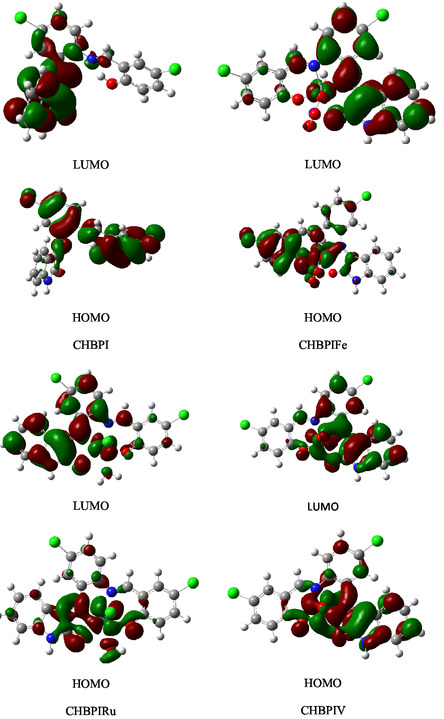
Visual representations of HOMO and LUMO for the examined ligand CHBPI and its corresponding iron, ruthenium, and vanadium oxide chelates: an analysis conducted with B3LYP/6‐311G(d, p) and B3LYP/6‐311‐LANL2DZ computational methods.

In order to comprehend the electron density and charge migration within a given chemical entity, one must meticulously scrutinize the spatial configuration of molecular orbitals and their corresponding energy levels. Figure 6 illustrates the HOMO and LUMO mapping diagrams for the CHBPI ligand along with its Fe, Ru, and VO complexes. The evaluation of the redox potentials of these substances is contingent upon the understanding of the energies associated with their HOMO and LUMO states.

The concept of chemical reactivity and molecular hardness or softness is significantly influenced by the energy gap, denoted as *E*_gap_, which refers to the disparity in energy levels between the (*E*_HOMO_) and the (*E*_LUMO_). This energy gap is a critical factor in determining how readily a molecule can undergo electronic transitions and participate in chemical reactions. Molecules characterized by smaller energy gaps (*E*_gap_) are typically more reactive and globally softer, allowing for smoother charge transfer and polarization to occur. This is in stark contrast to molecules with larger *E*_gap_ values, which tend to be harder and less reactive, necessitating a greater amount of energy to undergo similar processes. Table 6 compiles computed molecular properties, including total energy, energy gap (Egap), electron affinity, ionization potential, electronegativity, chemical hardness, chemical potential, and global softness (S). Collectively, these metrics offer critical insights into the reactivity patterns and softness characteristics exhibited by the analyzed compounds.

**TABLE 6:** Examine the comprehensive energy properties, including the total energy, the HOMO (highest occupied molecular orbital) and LUMO (lowest unoccupied molecular orbital) energy levels, the energy gap between these orbitals, ionization potential (I), electron affinity (A), absolute electronegativities (χ), overall hardness (χ), global softness (S), and chemical potential (V) for the CHBPI ligand and its chelates with iron, ruthenium, and vanadyl (VO) ions, using the computational approach of B3LYP/6‐311G(d, p) and B3LYP/6‐311G(d, p)‐LANL2DZ.

Parameter	Ligand (CHBPI)	CHBPIFe	CHBPIRu	CHBPIV
E_T_, a.u.	−2043.49	−2523.24	−2673.48	−2189.58
E_HOMO_, a.u.	−0.2240	−0.1843	−0.1701	−0.1608
E_LUMO_, a.u.	−0.1020	−0.1303	−0.1227	0.1201
E_g_, eV	3.2929	1.4692	1.2906	1.1097
I, eV	6.0951	5.0154	4.6287	4.3767
A, eV	2.8023	3.5462	3.3380	3.2670
χ, eV	4.4487	4.2808	3.9833	3.8219
η, eV	1.6464	0.7346	0.6453	0.5548
S, eV	0.3037	0.6807	0.7748	0.9012
V eV	−4.4487	−4.2808	−3.9834	−3.8219

Chelation modifies the electronic characteristics of Fe, Ru, and VO‐bound CHBPI complexes, evidenced by ionization potential (Ip) measurements. These complexes display destabilized highest occupied molecular orbital (HOMO) levels at energies of −0.1843 , −0.1701 , and −0.1608 eV respectively—significantly higher than the ligand's HOMO energy of −0.2240 eV. In contrast, chelation stabilizes their lowest unoccupied molecular orbital (LUMO) energies. The HOMO electron density primarily resides on oxygen/nitrogen atoms and phenyl rings, concentrated within the metal coordination sphere while excluding phenyl groups. Conversely, LUMO density distributes uniformly across metal cation regions in the chelates, avoiding phenyl rings entirely (Table 6). Reactivity analysis reveals narrower energy band gaps in these chelates compared to the free ligand. This reduced gap corresponds to lower chemical hardness (η) and elevated softness (S), indicating enhanced reactivity through facilitated polarization and electron transfer mechanisms.

#### 3.12.4 Molecular Electrostatic Potential (MESP)

**FIGURE 7:**
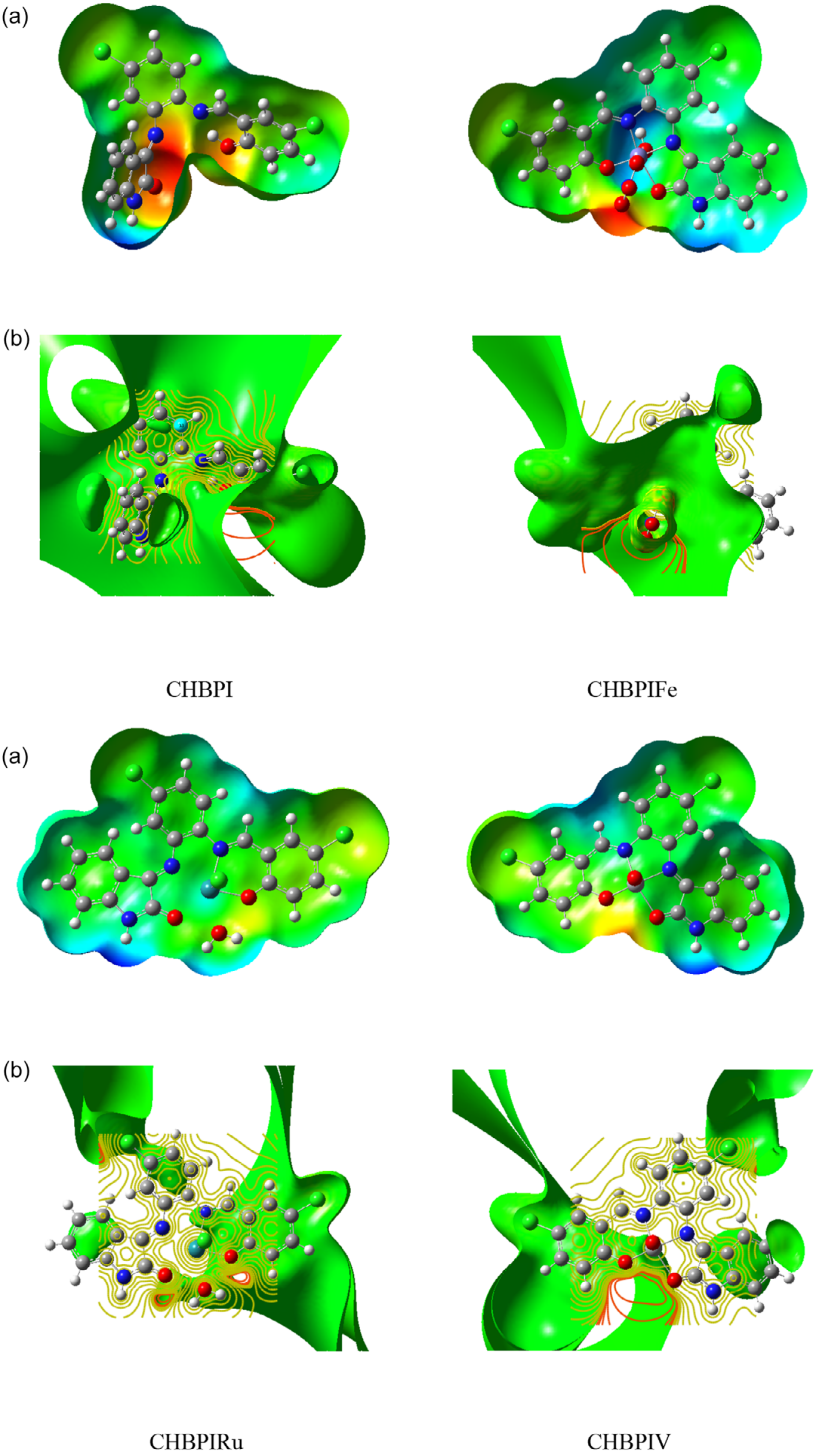
Examination of molecular electric potentials and corresponding electrostatic surface contour visualizations (a) and (b) for the investigated CHBPI ligand in combination with its iron, ruthenium, and vanadium oxo chelates utilizing B3LYP/6‐311G(d, p) and B3LYP/6‐311G(d, p)‐LANL2DZ theoretical levels.

The three‐dimensional representations of electrostatic potential (ESP) and molecular electrostatic potential (MESP) for the CHBPI ligand and its derived Fe, Ru, and CHBPIV chelates are presented in Figure 7. These plots were constructed based on the optimized structures obtained through B3LYP/6‐311G (d, p) and B3LYP/6‐311G(d, p)‐LANL2DZ computational methods. The evaluation of intrinsic charge distribution and potential sites for charge transfer is crucial for understanding the nature of chemical interactions such as electrophilic or nucleophilic attacks [73, 74]. The color scale utilized ranges from red, indicating negative potential, through orange and yellow to green, and then to blue, which signifies positive potential [75]. The visual analysis reveals that the ligand and its chelates exhibit red regions, which correspond to negative potential, primarily localized over the nitrogen and oxygen atoms. These atoms, due to their lone pair electrons, contribute significantly to the red coloration in the Fe, Ru, and CHBPIV complexes. Notably, the ligand's atoms N10, N11, O20, and O39 are encircled by a yellow halo, which suggests a moderately negative potential. In contrast, the blue color, indicative of positive potential, is predominantly observed around hydrogen and carbon atoms. The presence of negative electrostatic potential around the nitrogen and oxygen atoms in the chelates is noteworthy, as it signifies a high likelihood for these sites to be involved in electrophilic interactions. Conversely, the positive regions, which are mainly associated with hydrogen atoms, are less likely to participate in nucleophilic attacks. The data implies that as the negative electrostatic potential enhances at these sites, the probability of an electrophilic attack occurring also increases, which is consistent with the established principles governing chemical reactivity and the behavior of species with distinct charge characteristics.

The biological activity of the synthesized nanoscale compounds can be rationalized based on the calculated B3LYP functional descriptors. The HOMO–LUMO energy gap provides insight into molecular reactivity, where a smaller gap indicates higher charge transfer capability and enhanced interaction with biological targets, which may contribute to improved antimicrobial performance. In addition, the molecular electrostatic potential (MEP) surface analysis reveals the distribution of electron‐rich and electron‐deficient regions within the molecules, identifying potential sites for hydrogen bonding and electrostatic interactions with microbial cell components, thereby supporting the observed variation in biological activity.

#### 3.12.5 Natural Charge and Natural Population

Transition metal complexes incorporating isatin ligands at the nanoscale—specifically those involving VO(II), Fe(III), and Ru(III)—have attracted significant research attention owing to their exceptional nonlinear optical (NLO) characteristics. These properties are critical for cutting‐edge photonic and optoelectronic technologies. The enhanced NLO performance arises from two key factors: the extended π‐conjugation within the isatin framework and the specific geometry imposed by metal coordination. Nanoscale structuring further intensifies optical responses through heightened surface interactions and quantum confinement, boosting polarizability and third‐order nonlinear susceptibility. Among these metals, Ru(III) complexes demonstrate superior NLO activity due to optimized electron transfer dynamics between the metal center and ligands. These attributes make such complexes highly suitable for optical switching, sensing applications, and frequency‐conversion devices. Charge distribution analysis across atomic sites provides deeper insight into the electrical behavior of these systems. Computational data (Table 5) reveal that the effective charge on Fe, Ru, and VO^2+^ ions falls below their formal oxidation states (+3 or + 4). This reduction stems from electron donation from coordinating atoms—notably N10, N11, O20, and O39—in the ligand structure. The ligand atoms function as primary electron donors within these complexes, displaying the highest concentration of negatively charged sites. Analysis of atomic natural charges reveals significant reductions for Fe, Ru, and VO metal ions—specifically 0.430, 0.552, and 0.750 respectively. This phenomenon correlates with their 3d/4d orbital configurations: Fe (3d^6.86^), Ru (4d^6.86^), and VO (3d^3.71^), indicating diminished electropositivity relative to their formal oxidation states. Supplemental data (Table S3) further demonstrates substantial electron density transfer from ligands to metal centers: Fe acquires 2.57e (yielding 3d^6.45^), Ru gains 2.448e (resulting in 4d^8.71^), and VO accumulates 3.25e (producing 3d^9.99^). Such electron redistribution underscores the ligands’ critical role in stabilizing metal ions and shaping the chelates’ electronic architecture.

#### 3.12.6 Nonlinear Optical Properties (NLO)

The arrangement of atomic charges within a molecule has a profound impact on both the magnitude and direction of the resulting moments, which are essential in determining the molecule's interaction with electromagnetic fields. Nonlinear optical (NLO) compounds are of particular significance in the realms of optical data storage, computing, and information processing due to their exceptional properties, sparking a high level of interest and research [76, 77]. Table 7 showcases the computed total static polarizabilities, dipole moments, and hyperpolarizabilities of the CHBPI ligand and its complexes with Fe, Ru, and VO metals. These values were obtained using theoretical models and then compared to empirical data to evaluate their accuracy [78, 79]. Since experimental NLO values for these specific compounds are not readily available, Urea was utilized as a reference to provide context for the results [80]. It is important to note that while these findings offer valuable insights, they may not be as conclusive or authoritative as the data presented in earlier studies on similar molecules [78, 81]. Polarizabilities and first‐order hyperpolarizabilities were quantified in atomic units (au) and then converted for practical application by using the appropriate ratios of 8.6393 × 10^−33^ esu for (β) values and 0.1482 × 10^−24^ esu for (α) values. The data in Table 7 reveals that all compounds exhibit polar characteristics, as indicated by their substantial dipole moments (μ). The polarity sequence, from highest to lowest, is as follows: CHBPIFe, CHBPIRu, CHBPI, and CHBPIV, with all dipole moments exceeding that of Urea. The first‐order hyperpolarizability is a pivotal factor in assessing NLO systems, and the β parameter range indicates that the CHBPI ligand possesses a value tenfold greater than Urea. Notably, the CHBPIFe chelate exhibits a 17‐fold increase over Urea, while the CHBPIRu chelate shows a 22‐fold enhancement, and the CHBPIV chelate a more modest eleven‐fold improvement. This suggests that the CHBPI ligand and its metal chelates, particularly CHBPIFe and CHBPIRu, are highly promising candidates for the development of advanced NLO materials. The variations in hyperpolarizability among the chelates highlight the substantial impact of the metal centers on the NLO properties.

**TABLE 7:** The total static dipole moment (μ), average polarizability (<α>), polarizability anisotropy (Δα), and first hyperpolarizability order (<β>) of the CHBPI ligand as well as its complexes with Fe, Ru, and VO were determined using computational methods.

Property	Urea	Ligand CHBPI	CHBPIFe	CHBPIRu	CHBPIV
µ, D	1.3197	5.3292	10.8678	8.4630	5.3245
XX, a.u.	—	−177.9969	−201.0759	−196.9184	−183.1648
YY	—	−155.2405	−199.2064	−179.4154	−170.0838
ZZ	—	−183.1553	−218.2887	−229.3406	−209.4517
XY	—	15.6218	−25.8764	10.9167	−17.0576
XZ	—	−11.6148	11.2523	1.2068	−2.9334
YZ	—	−3.3854	12.1636	−0.4947	1.2447
<α> esu	—	−2.5510 × 10^−23^	−3.0557 × 10^−23^	−2.9920 × 10^−23^	−2.7797 × 10^−23^
Δα, esu	—	3.8127 × 10^−24^	2.7002 × 10^−24^	6.5023 × 10^−24^	5.1466 × 10^−24^
XXX	—	−70.9273	270.1396	−272.6171	139.4078
XXY	—	−100.2338	−143.5695	−177.5022	−106.8675
XYY	—	−29.765	40.0358	7.9742	7.2711
YYY	—	−87.4272	−60.3843	−214.8731	−87.8354
XXZ	—	−31.8268	4.6527	−11.366	−8.798
XYZ	—	28.3863	−10.3964	5.7957	2.2596
YYZ	—	21.8012	−34.7529	−5.1933	1.5874
XZZ	—	−17.4173	−12.747	5.5635	5.7763
YZZ	—	11.7777	4.5041	−23.8875	0.3979
ZZZ	—	44.4075	−97.9068	−8.2659	4.3768
<β>, esu	0.1947 × 10^−30^	1.8543 × 10^−30^	3.2855 × 10^−30^	4.2413 × 10^−30^	2.1338 × 10^−30^

### 3.13 Molecular Docking on Antimicrobial and Breast Cancer Activity

The functioning of antibacterial medications typically encompasses the hindrance of cellular wall synthesis, the suppression of protein synthesis, the inhibition of nucleic acid (DNA) production, and anti‐metabolic activities [82]. Molecular docking analyses have been conducted to assess the proficiency of these inhibitors, using the binding pocket residues from PDB (Protein Data Bank) structures for both bacterial and *breast cancer* targets (Table 4S). The study involved examining the binding profiles of the candidate substances with the protein targets from two bacterial species, namely *Candida albicans* (PDB ID: 3QLW) and *Micrococcus luteus* (PDB ID: 3IF5), as well as with *breast cancer* cells (PDB ID: 3HB5) and their standard antibiotics (fluconazole, ofloxacin, and cisplatin). The molecular docking process provided insights into the binding free energy and interactions between the test compounds and the bacterial protein receptors. The outcomes of these interactions are presented in Table S4. The analysis included 2D and 3D visualizations of the docking interactions, with hydrogen bonds being denoted by dashed lines in Figure 8. It was found that the bonding interfaces between the synthesized compounds, their standard antibiotics and the bacterial proteins, as well as the breast cancer receptors, involved a combination of π‐hydrogen, hydrogen‐donor, hydrogen‐π, hydrogen acceptor, π–π, metal, and ionic interactions. These various bonding types suggest robust stability in the docked complexes, which is crucial for effective inhibition. The data from Table 4S reveals that the CHBPIV‐3IF5 chelate exhibits the most negative binding energy score at −7.02 kcal/mol, indicating a stronger interaction with the bacterial proteins compared to the 3HL and 3HB5 protein receptors. Furthermore, the CHBPI‐3HB5 complex demonstrates higher inhibitory effectiveness against breast cancer cells than its counterparts CHBPIFe‐3HB5, CHBPIRu‐3HB5, and CHBPIV‐3HB5, as evidenced by its superior binding affinity. The order of binding affinity for the iron, ruthenium, and vanadium‐containing CHBPI chelates with the breast cancer protein receptor is CHBPIV‐3HB5 > CHBPI‐3HB5, which suggests that the vanadium derivative may have the most potent inhibitory effect. Figure 8 shows that the bonding interfaces between the standard antibiotics and the bacterial proteins, as well as the breast cancer receptors, involved a combination of π‐hydrogen and hydrogen acceptor. The data from Table S4 indicates that the standard antibiotics exhibit a less negative binding energy score than the prepared compounds, representing a lower interaction with the bacterial proteins compared to the 3HL and 3HB5, 3QLW, and 3IF5 protein receptors, indicating highly effective inhibition.

FIGURE 8Exploratory analysis of molecular docking experiments illustrating the binding affinity of probed ligand CHBPI, its corresponding chelates and standard antibiotics, with key target sites in breast cancer receptor (3HB5), *C* *andida albicans* (3QLW), and *M* *icrococcus luteus* protein structures.

**Figure:**
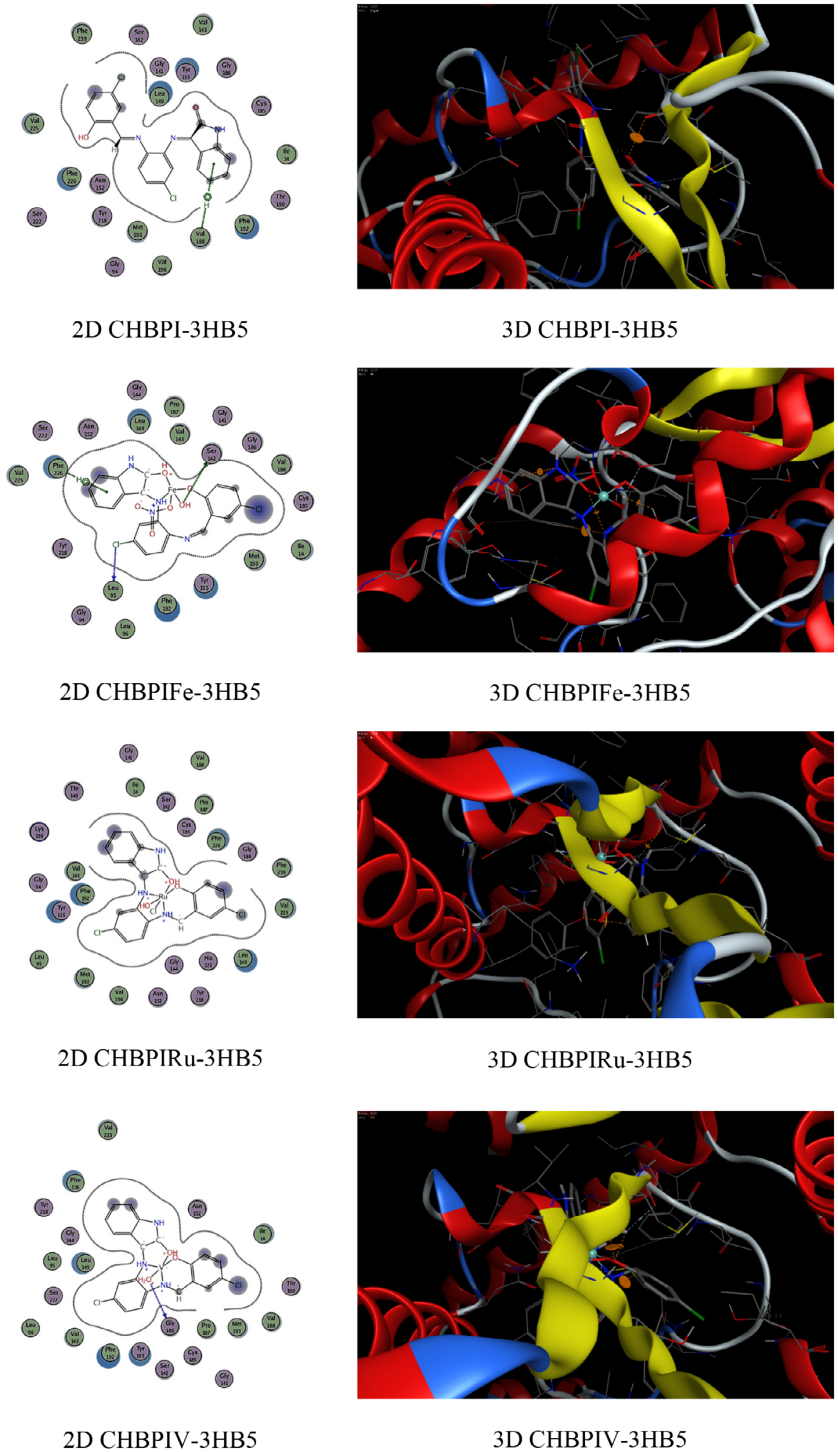

**Figure:**
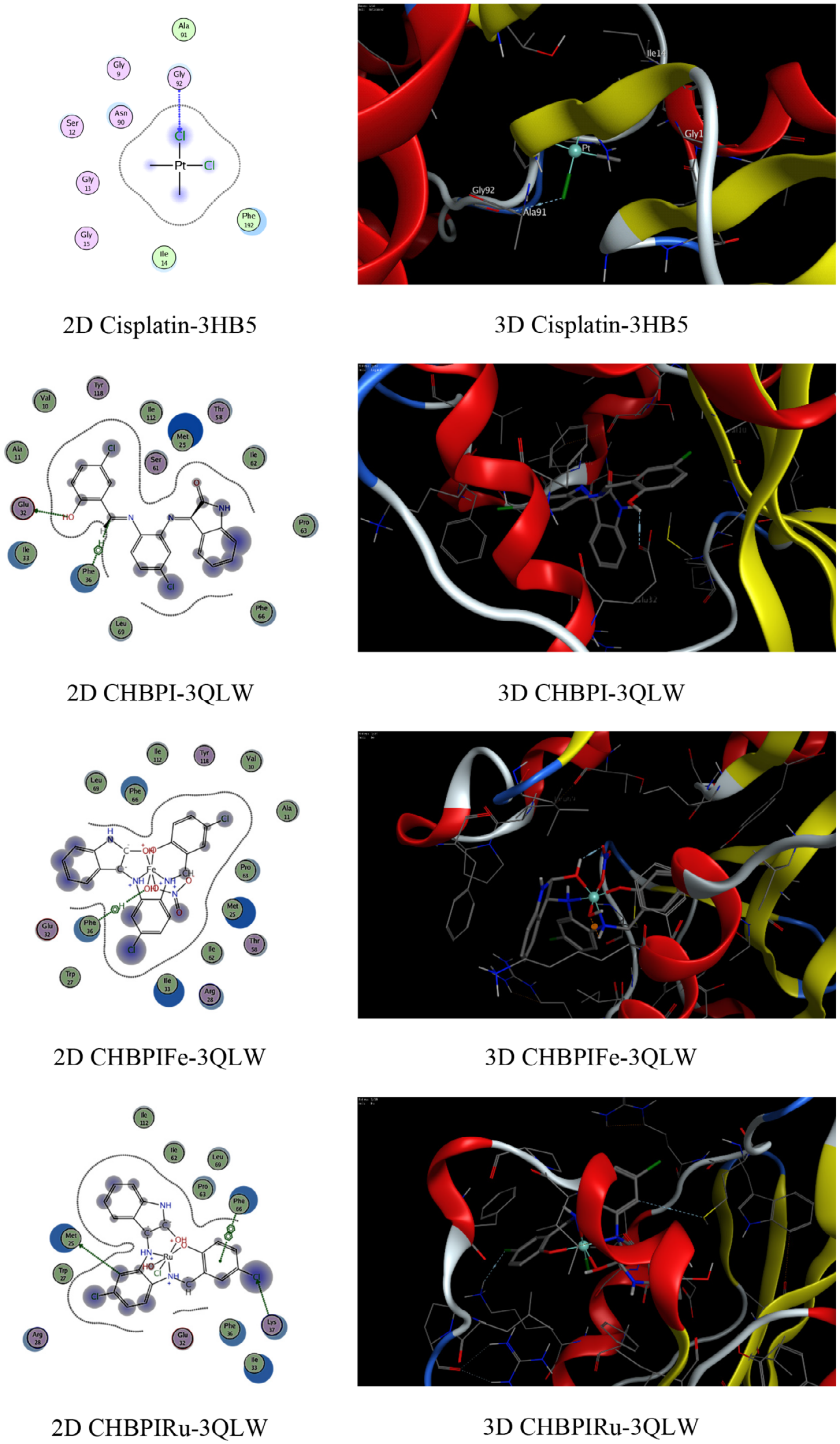

**Figure:**
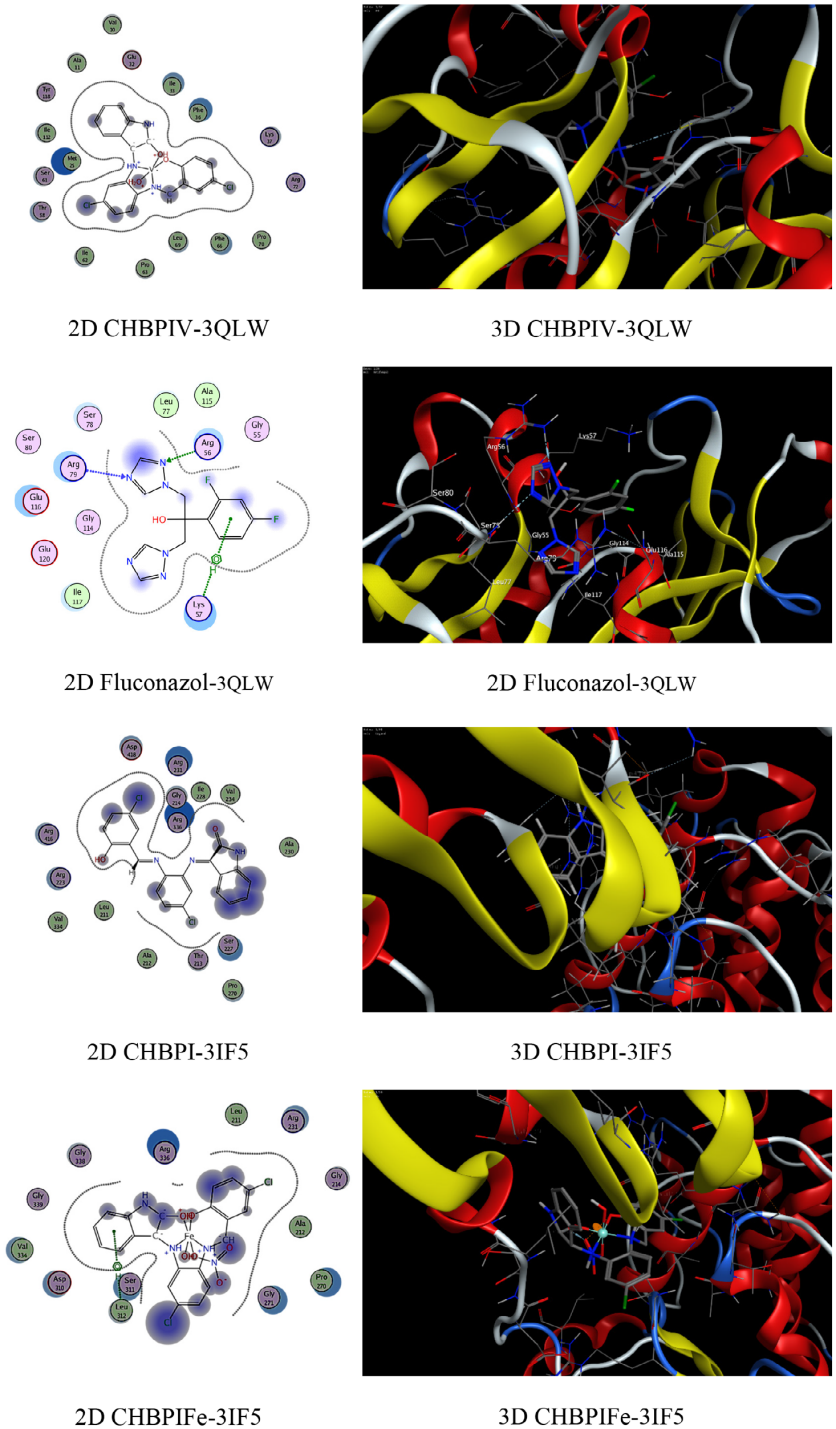

**Figure:**
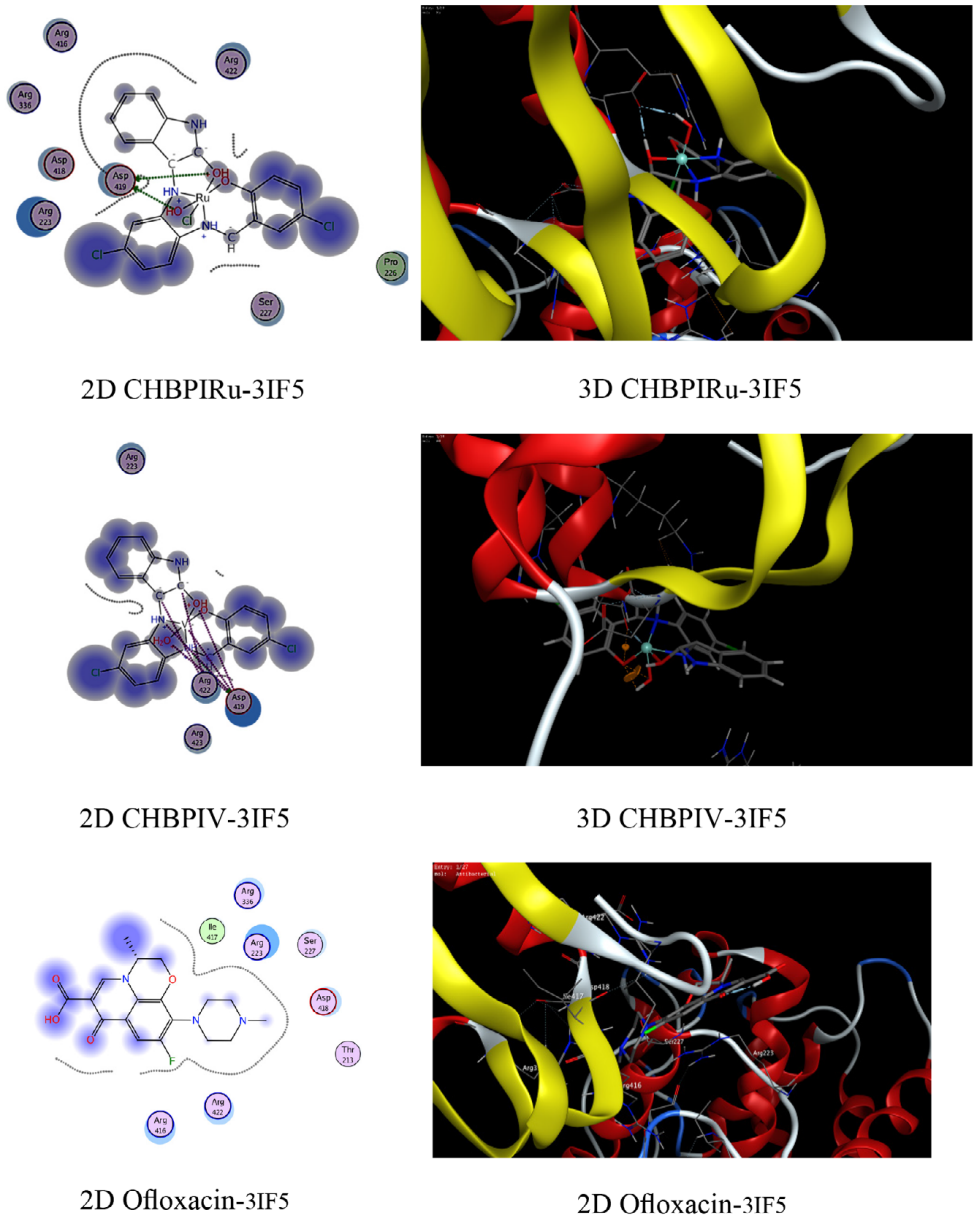

### 3.14 Biological Activity

#### 3.14.1 Antimicrobial Activity

**FIGURE 9:**
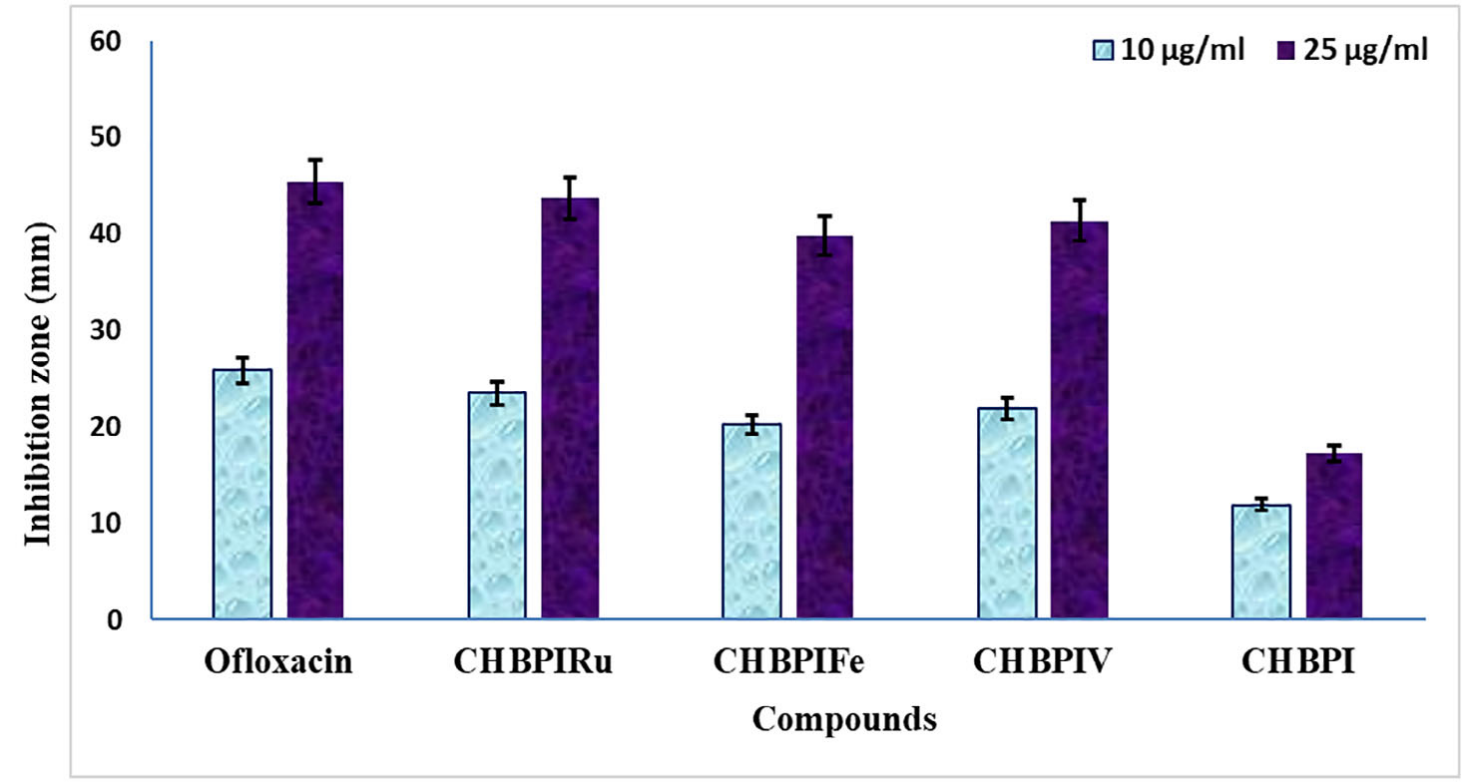
Histogram showing the comparative antibacterial activities of CHBPI and its complexes agonist *M. luteus* at a concentration of 10 and 25 µg ml^−1^.

**FIGURE 10:**
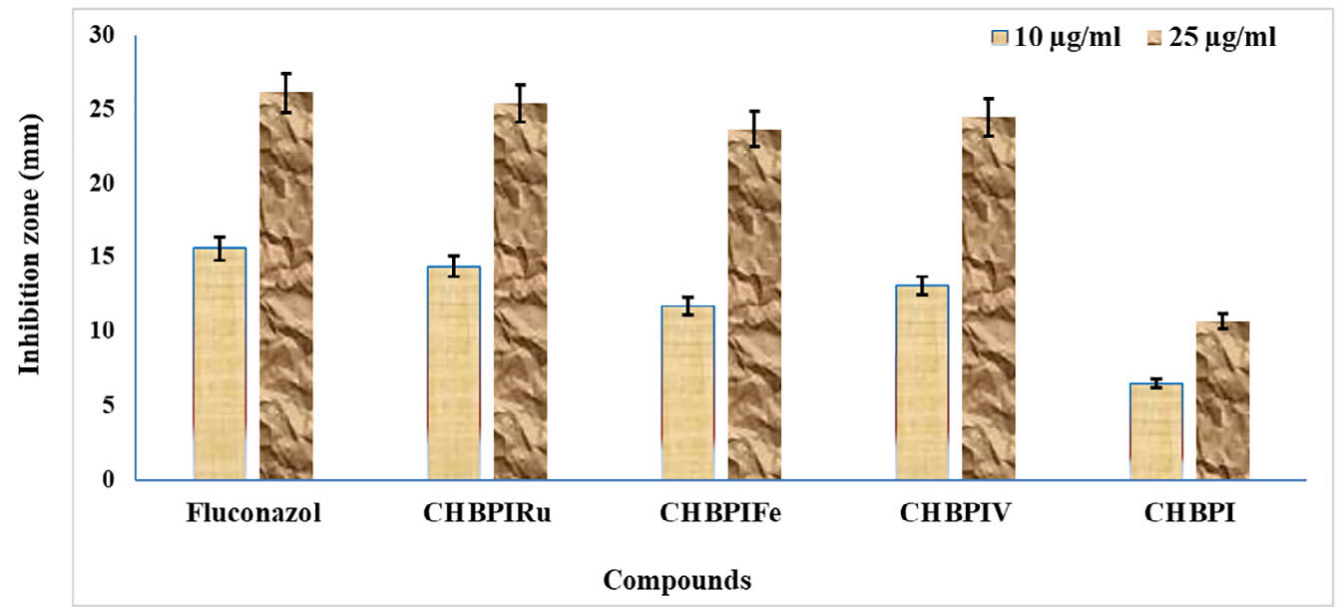
Histogram showing the comparative antifungal activities of CHBPI and its complexes against *A. flavus* at a concentration of 10 and 25 µg ml^−1^.

The antimicrobial assessment findings have been consolidated in Figure 9, 10, and Tables 5S, 6S. The CHBPI Schiff base ligand, along with its derived complexes, underwent examination for their efficacy against a pair of Gram‐negative bacterial strains, *E. coli* and *S. Marcescens*, as well as a single Gram‐positive bacterium, *M. Luteus*. Additionally, antifungal properties were evaluated by challenging these substances with three species of pathogenic fungi: *F. oxysporum*, *C. albicans*, and *A. flavus*. The study revealed that both the ligand and its complexes exerted a spectrum of inhibitory impacts on the proliferation of the chosen microbes. Notable sensitivity to the synthesized complexes was observed in *M. luteus* (as depicted in Figure 9) and A. flavus (illustrated in Figure 10).

Upon analyzing the complexes’ activities in relation to the parent ligand, it appeared that the complexes generally demonstrated superior antibacterial and antifungal potency under identical experimental parameters, with the Ru(III) complex emerging as the most efficacious. This enhanced performance may be ascribed to disparities in geometric structure and steric hindrance, which likely facilitate the complexes’ entry into and subsequent suppression of microbial cells.

Indeed, there appears to be a direct relationship between the stability of certain prepared complexes and their efficacy in combating various microorganisms. The stability constants of the complexes, which have been established through calculation, reveal an ascending order of stability as follows: CHBPIV < CHBPIFe < CHBPIRu. This suggests that the stability of the CHBPI isatin complexes may be associated with their biological potency against diverse pathogenic microbial strains. The observed increase in stability in the Ru(III) complex could potentially be linked to a heightened biological activity. The explanation for the remarkable antimicrobial properties of these complexes can be found in the Overtone's concept and chelation theory [9, 15, 27, 32, 33, 34, 83]. These theories posit that upon chelation, the polarity of the metal ion diminishes significantly due to the interaction between the ligand's orbitals and the metal ion's positive charge. This interaction leads to an extensive delocalization of p‐electrons across the entire chelating ring structure, thereby enhancing the complex's ability to penetrate the lipid membranes of microorganisms and inhibit the function of their enzymes by obstructing metal binding sites.

Several additional factors might also contribute to the augmented activity of these compounds, such as their solubility characteristics, electrical conductivity properties, and the metal–ligand bond lengths [84, 85]. The efficacy of the CHBPI Schiff base ligand and its complexes in inhibiting microbial growth was determined by employing a serial dilution technique, and the resulting data are presented in Table 8.

**TABLE 8:** The results of the minimum inhibition concentration (MIC) of the ligand and its metal complexes against the different strains of bacteria and fungi.

Compound	(MIC) minimum Inhibition concentration µg/mL					
	**Bacteria**			***Fungi***		
	***E.*** ***coli*** ***(‐ve)***	***S.*** ***marcescence*** ***(‐ve)***	***M.*** ***luteus (+ve)***	***F.*** ***oxysporum***	***C.*** ***albicans***	A. *flavus*
**CHBPI**	7.50	7.00	6.50	6.50	6.00	8.25
**CHBPIV**	3.00	2.25	1.75	2.75	2.25	2.50
**CHBPIFe**	3.50	1.50	2.00	3.25	2.50	2.75
**CHBPIRu**	2.75	2.00	1.50	2.50	1.75	2.25
**Ofloxacin**	2.50	1.75	1.25			
**Fluconazol**				2.00	1.50	2.25

The observed variations in minimum inhibitory concentration (MIC) values against Gram‐positive and Gram‐negative bacterial strains can be rationalized in terms of structure–activity relationships (SAR) of the synthesized nanoscale metal complexes. The enhanced antimicrobial activity upon complexation is primarily attributed to increased lipophilicity, which facilitates penetration through the lipid bilayer of microbial cell membranes and improves interaction with intracellular targets [9, 15, 32, 33, 34]. In Gram‐negative bacteria, the outer membrane acts as an additional barrier; however, the chelation of metal ions reduces the polarity of the ligand system, thereby enhancing membrane permeability and leading to improved antimicrobial efficacy [86, 87, 88]. In contrast, Gram‐positive bacteria possess a thicker peptidoglycan layer, which influences the degree of interaction depending on the nature of the metal center and ligand framework. Furthermore, the electronic properties derived from the B3LYP functional calculations, including HOMO–LUMO energy gap and charge distribution, also play a significant role in biological activity. Compounds with lower energy gaps and enhanced charge delocalization exhibit stronger interactions with microbial biomolecules, contributing to lower MIC values and higher antimicrobial potency.

Furthermore, an analysis of the activity index, as outlined in Table 9, serves to substantiate the relative antimicrobial capabilities of the synthesized compounds.

**TABLE 9:** Antimicrobial activity index (%) of the ligand and its complexes.

Compound	Activity index, %					
	Bacteria			**Fungi**		
	*E. coli* (‐ve)	***S.*** ***marcescence*** ***(‐ve)***	***M.*** ***luteus (+ve)***	***F.*** ***oxysporum***	*C. albicans*	*A.* *flavus*
**CHBPI**	34.56	44.11	37.88	36.99	35.23	40.99
**CHBPIV**	89.24	89.07	91.07	90.34	91.49	93.67
**CHBPIFe**	79.54	84.59	87.77	86.03	87.24	90.61
**CHBPIRu**	94.35	94.25	96.14	94.50	96.71	97.31

### 3.15 Anticancer

**FIGURE 11:**
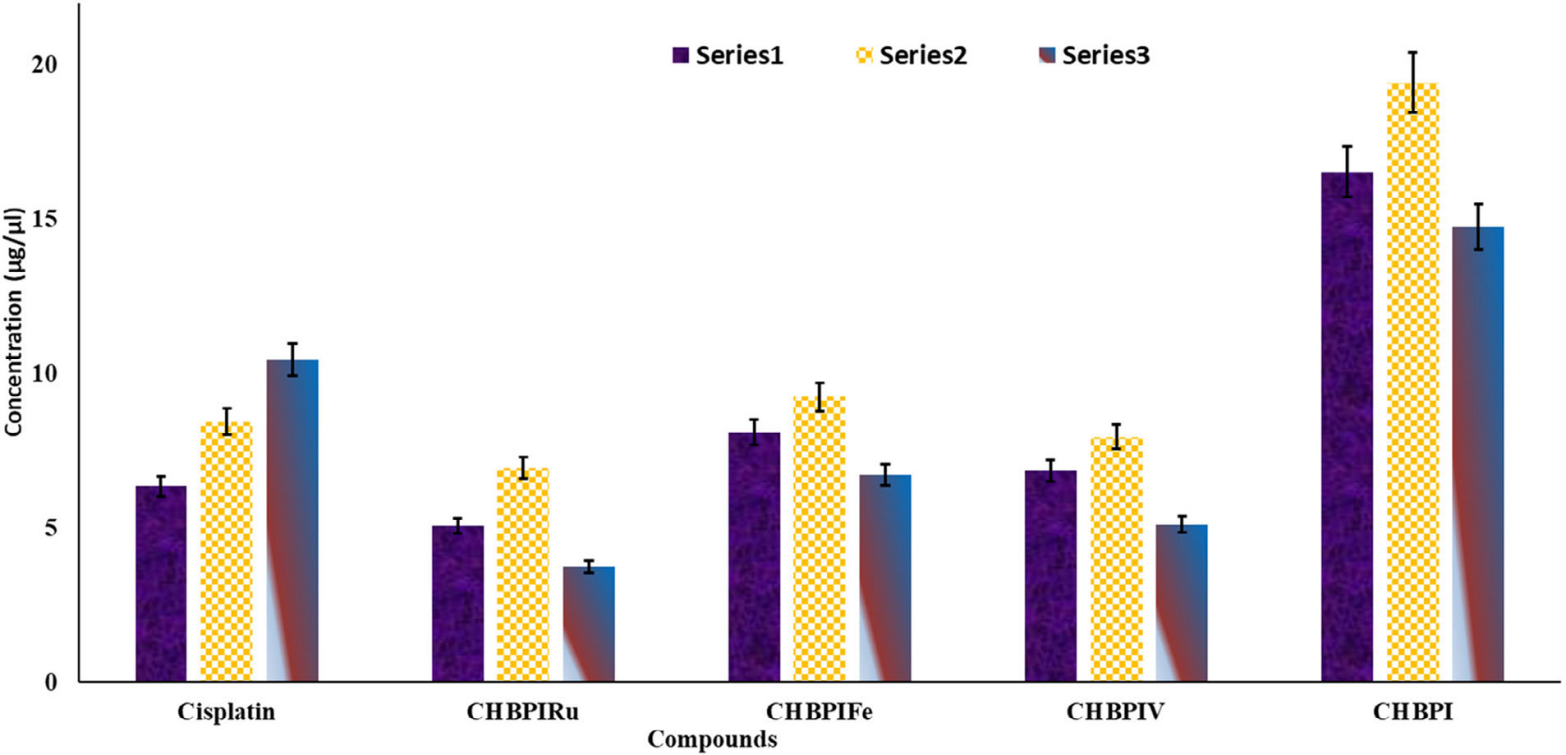
IC_50_ values for CHBPI, its metal complexes and cisplatin drug against (1) Hep‐G2, (2) HCT‐116, and (3) MCF‐7 cell lines.

The biological impact of CHBPI complexes on Hep‐G2 (hepatic cancer), MCF‐7 (breast cancer), and HCT‐116 (colonic cancer) cell lines was assessed for cytotoxic properties. The IC_50_ values, presented in Figure 11 and Table S7, revealed that the majority of these complexes exhibited enhanced cytotoxicity in comparison to the conventional Cisplatin. This suggests that the specific metal ion involved and the location of complexation within the molecule significantly influence the biological activity, aligning with Tweedy's chelation theory [89, 90]. Further analysis indicated that CHBPIV, CHBPIFe, and CHBPIRu complexes show remarkable cytotoxicity against HCT‐116 cells, with IC_50_ values ranging from 6.95 to 9.25  μg/μL, MCF‐7 cells with IC_50_ values of 3.75–6.72 μg/μL, and Hep‐G2 cells with IC_50_ values of 5.07–8.10 μg/μL. The CHBPIRu complex demonstrated the most potent effect on HCT‐116 cells with an IC_50_ of 6.95 μg/μL, while on MCF‐7 cells, it was the highest with an IC_50_ of 3.75 μg/μL. For Hep‐G2 cells, CHBPIRu had the best performance with an IC_50_ of 5.07 μg/μL. In all instances, these complexes surpassed the cytotoxic activity of the CHBPI ligand alone, pointing to a synergistic enhancement upon metal coordination. The observed increase in potency can be explained by the metal's positive charge, which is known to augment the acidity of the coordinated ligand, thereby strengthening its ability to form hydrogen bonds [89]. This enhancement of biological activity may stem from the metal's role in facilitating DNA binding interactions [86]. Additionally, studies by Gaetke and Chow [90] suggest that metals can mitigate oxidative tissue damage through a mechanism akin to the Fenton reaction, which could further contribute to the observed cytotoxicity. The alteration in coordination sites and metal ion identity within the CHBPI complexes thus plays a crucial role in modulating their interaction with cancer cells and, consequently, their therapeutic efficacy.

### 3.16 Examination of DPPH Radical Scavenging Efficiency

Several research studies have utilized the capacity of substances or molecules to neutralize the DPPH radical as a reliable and accurate method to assess their in vitro antioxidant properties [91]. This is typically determined by examining the reduction in DPPH's molar absorptivity at 517 nm following interactions with the test compound. The antioxidant effect on DPPH radicals can be ascribed to the substance's ability to donate hydrogen atoms or serve as direct radical scavengers [92]. The interaction can be conceptualized as a process where the antioxidant (H‐D) reacts with the DPPH radical:

(DPPH) + (H‐D) (Purple) → DPPH‐H + (D)(Yellow)(DPPH) + (H‐D) (Purple) → DPPH‐H + (D)(Yellow)

**FIGURE 12:**
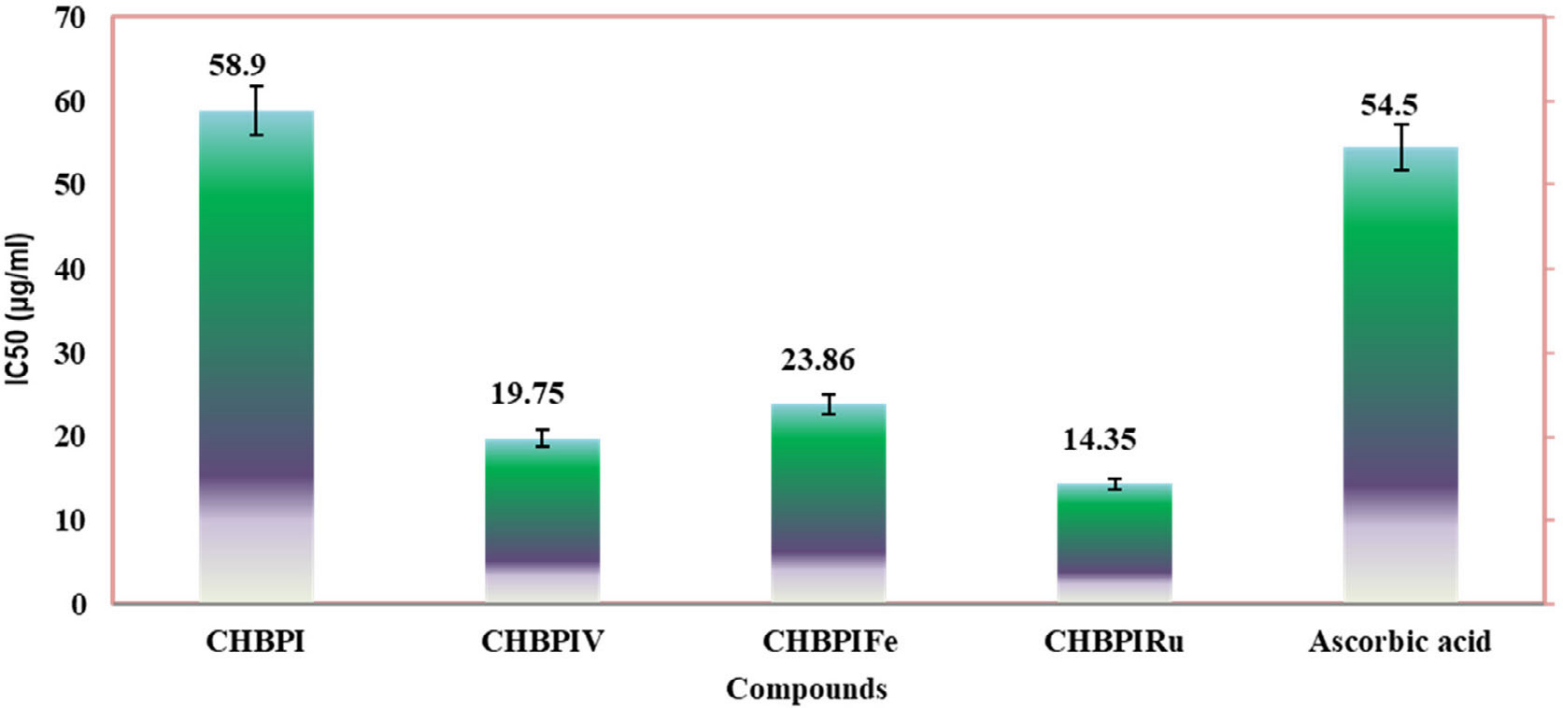
DPPH suppression by the investigated CHBPI complexes.

After interacting with an antioxidant, DPPH radicals are converted into less stable free radicals, resulting in a decrease in absorbance due to the formation of DPPH‐H. The scavenging efficiency is often quantified by the extent of discoloration, reflecting the antioxidant's hydrogen donating or radical scavenging prowess. When analyzing the dose–response curves for DPPH activity, such as the one presented in Table S8 and Figure 12, it is evident that the complexes formed between CHBPI and transition metals like VO(II), Fe(III), and Ru(III) exhibit enhanced antioxidant effects compared with the free CHBPI ligand. The uncomplexed CHBPI ligand had an IC_50_ value of 58.9% at the screened dosage of 100 g/mL. Upon complexation with the metals, the IC_50_ values notably decreased, at 19.75%, 23.86%, and 14.35% for CHBPIV, CHBPIFe, and CHBPIRu, respectively. This suggests that the formation of these metal complexes significantly enhances the antioxidant activity of the CHBPI ligands, potentially due to changes in their electronic structures or increased stability in the presence of the metal ions.

## 4 Conclusion

The prpearation and characterization of three novel complexes derived from the CHBPI ligand with VO^2+^, Fe^3+^, and Ru^3+^ cations were successfully achieved using an array of spectroscopic and analytical techniques. These complexes were further validated through B3LYP functional calculations, which suggested octahedral geometry for CHBPIRu and CHBPIFe, and square pyramidal geometry for CHBPIV. The spectroscopic analysis confirmed a 1:1 (M:L) stoichiometric ratio, with the ligand acting as a neutral tetradentate donor, and the complexes exhibited excellent stability over a pH range of 4–11.

Quantitatively, the biological evaluation demonstrated significant activity differences among the complexes, where CHBPIRu showed the lowest MIC values and highest cytotoxic efficiency against tested microbial strains and cancer cell lines, indicating superior bioactivity. These results are consistent with docking binding energy calculations, which further supported stronger ligand–target interactions for CHBPIRu compared with CHBPIV and CHBPIFe. In addition, antioxidant assays revealed that all complexes exhibited higher DPPH radical scavenging activity than ascorbic acid, confirming their strong redox potential.

The structure–activity relationship observed across experimental and theoretical data highlights the critical role of metal ion identity in modulating electronic structure, binding affinity, and biological response. In particular, enhanced charge transfer properties and reduced energy gaps correlate well with improved biological performance.

Future work will focus on expanding quantitative kinetic modeling, detailed in vivo investigations, and further optimization of metal–ligand frameworks to enhance selectivity and efficacy. These nanoscale complexes also show promising potential for future applications in antimicrobial drug development, anticancer therapy, and multifunctional antioxidant agents in biomedical and pharmaceutical fields.

## Author Contributions

**R.A. El‐Kasaby**: investigation, methodology, resources, writing – original draft, project administration, writing – review and editing, supervision. **Eida S. Al‐Farraj**: conceptualization, methodology, data curation, writing – original draft, visualization, investigation, project administration, writing – review and editing, funding. **Senem Akkoc**: investigation, writing – review and editing. **Amal H. Alsehli**: conceptualization, methodology, data curation, writing – original draft, visualization. **Maher Fathalla**: conceptualization, methodology, data curation, writing – original draft, visualization. **Samir A. Abdel‐Latif**: conceptualization, methodology, data curation, writing – original draft, visualization. **Mashael A. Alghamdi**: formal analysis, investigation, resources, data curation, administration, funding. **Ahmed M. Abu‐Dief**: conceptualization, resources, methodology, data curation, writing – original draft, visualization, investigation, project administration, writing – review and editing, supervision.

## Funding

This work was supported by the Imam Mohammad Ibn Saud Islamic University (grant IMSIU‐DDRSP2601).

## Conflicts of Interest

The authors declare no conflicts of interest.

## Supporting information

Supplementary Material

## Data Availability

Data will be available on request by the authors.

## References

[1] A. M. Abu‐Dief , H. M. El‐Sagher , and M. R. Shehata , “Fabrication, Spectroscopic Characterization, Calf Thymus DNA Binding Investigation, Antioxidant and Anticancer Activities of Some Antibiotic Azomethine Cu (II), Pd (II), Zn (II) and Cr (III) Complexes,” Applied Organometallic Chemistry 33 (2019): e4943.

[2] A. M. Abu‐Dief , M. R. Shehata , A. E. Hassan , et al., “Development of Some Novel Hydrophilic Schiff Base Complexes: Synthesis, Spectroscopic Characterization, and DFT Calculation DNA‐Binding and Biomedical Studies Supported by Molecular Docking Approach,” Applied Organometallic Chemistry 39 (2025): e70075.

[3] A. M. Abu‐Dief , M. A. Said , O. Elhady , et al., “Design, Structural Inspection of Some New Metal Chelates Based on Benzothiazol‐Pyrimidin‐2‐Ylidene Ligand: Biomedical Studies and Molecular Docking Approach,” Inorganic Chemistry Communications 158 (2023): 111587.

[4] K. D. Khalil , A. H. Bashal , T. Habeeb , R. Kebeish , and A. M. Abu‐Dief , “Multifunctional Lanthanum Oxide‐Doped Carboxymethyl Cellulose Nanocomposites: A Promising Approach for Antimicrobial and Targeted Anticancer Applications,” International Journal of Biological Macromolecules 283 (2024): 137495. 39528180 10.1016/j.ijbiomac.2024.137495

[5] W. H. Alsaedi , W. S. Mohamed , H. A. Qasem , et al., “Fabrication of CuO/PdO Nanocomposites for Biomedical Applications,” Inorganic Chemistry Communications 170 (2024): 113166.

[6] W. H. Alsaedi , A. Aljuhani , M. Alahmadi , et al., “Fabrication of a Novel ZnO/Lu2O3 Nanomaterial for the Photocatalytic Disposal of Methylene Blue Dye under Solar Cell Illumination,” Journal of Materials Science: Materials in Electronics 36, no. 5 (2025): 327.

[7] S. Hosny , M. R. Shehata , S. A. Aly , et al., “Designing of Novel Nano‐Sized Coordination Compounds Based on Spinacia Oleracea Extract: Synthesis, Structural Characterization, Molecular Docking, Computational Calculations, and Biomedical Applications,” Inorganic Chemistry Communications 160 (2024): 111994.

[8] H. Escobar‐Sánchez , C. C. Pardo , N. Benito , J. Hernández‐Montelongo , I. Nancucheo , and G. Recio‐Sánchez , “Advances in Synthesis of Transition‐Metal‐Based Nanoparticles and Their Bioapplications,” International Journal of Molecular Sciences 24, no. 22 (2023): 16489. 38003680 10.3390/ijms242216489 PMC10671710

[9] A. M. Abu‐Dief , M. R. Shehata , A. E. Hassan , et al., “Tailoring of Novel Water Soluble Pd (II), Cu (II), Fe (III) and VO (II) Chelates Based on 4‐[(5‐Bromo‐2‐Hydroxy‐Benzylidene)‐Amino]‐Benzenesulfonate Ligand: Synthesis, Spectral Investigations, DNA Interaction and Pharmaceutical Applications Supported by Molecular Docking Approach,” Journal of Molecular Structure 1334 (2025): 141780.

[10] S. Y. Lee , C. Y. Kim , and T. G. Nam , “Ruthenium Complexes as Anticancer Agents: A Brief History and Perspectives,” Drug Design, Development and Therapy 14 (2020): 5375–5392. 33299303 10.2147/DDDT.S275007 PMC7721113

[11] I. Omar , E. S. Al‐Farraj , M. Abdel‐Hameed , et al., “Integrated Experimental and Theoretical Investigation of VO (II), Ni (II), and Ru (III) Nanocomplexes Derived from a Tetradentate Anthraquinone Ligand: Sonochemical Synthesis and Therapeutic Assessment,” Journal of Molecular Structure 1353 (2026): 144779.

[12] A. M. Abu‐Dief , M. R. Shehata , A. E. Hassan , et al., “Exploring, Anthranilic Azomethine Complexes: From Synthesis, Spectroscopic, Solution and Theoretical Studies to DNA Interaction and Biomedical Prospects,” Journal of Molecular Structure 1341 (2025): 142571.

[13] R. Kakkar , “Isatin and Its Derivatives: A Survey of Recent Syntheses, Reactions, and Applications,” Medchemcomm 15, no. 10 (2019): 351–368. 10.1039/c8md00585k PMC6438150 30996856

[14] R. S. Cheke , V. M. Patil , S. D. Firke , et al., “Therapeutic Outcomes of Isatin and Its Derivatives against Multiple Diseases: Recent Developments in Drug Discovery,“ Pharmaceuticals (Basel) 15, no. 3 (2022): 272. 35337070 10.3390/ph15030272 PMC8950263

[15] A. A. Al‐Shamry , M. M. Khalaf , H. M. A. El‐Lateef , et al., “Development of New Azomethine Metal Chelates Derived from Isatin: DFT and Pharmaceutical Studies,” Materials 16, no. 1 (2023): 83. 10.3390/ma16010083 PMC9821024 36614421

[16] R. A. El‐Kasaby , E. S. Al‐Farraj , A. Abdou , and A. M. Abu‐Dief , “Synthesis, Spectral Analysis, Physicochemical Investigation and Biomedical Potential of Some Novel Cu (II), Ru (III) and VO (II) Complexes with Anthraquinone‐Based Schiff Base Supported by DFT and Molecular Docking Insights,” Journal of Molecular Structure 2025 (1345): 143010.

[17] T. J. Mason , “Sonochemistry: Current Uses and Future Prospects in Synthesis,” Ultrasonics Sonochemistry 58 (2019): 104602.

[18] M. Kamali , M. E. V. Costa , G. Otero‐Irurueta , and I. Capela , “Ultrasonic Irradiation as a Green Production Route for Coupling Crystallinity and High Specific Surface Area in Iron Nanomaterials,” Journal of Cleaner Production 211 (2019): 185–197.

[19] H. Muslu , Z. Kalaycıoğlu , T. Erdoğan , A. Gölcü , and F. Bedia Erim , “Synthesis, Characterization, Anti‐Inflammatory Evaluation, Molecular Docking and Density Functional Theory Studies of Metal Based Drug Candidate Molecules of Tenoxicam,” Results in Chemistry 3 (2021): 100111.

[20] S. Saikia and M. Bordoloi , “Molecular Docking: Challenges, Advances and Its use in Drug Discovery Perspective,” Current Drug Targets 20, no. 5 (2019): 501–521. 30360733 10.2174/1389450119666181022153016

[21] M. A. E. A. A. El‐Remaily., A. E. T. Nady., A. Alzahrani , et al., “Tailoring of Novel Ru (III) and Cr (III) Salen Complexes as Catalysts for a Sustainable and Green Synthesis of Dihydro‐Tetrazolo [1,5‐a] Thiazolo [4,5‐d] Pyrimidin‐6‐Yl Morpholine: Experimental and Theoretical Approaches,” Applied Organometallic Chemistry 39 (2025): e7879.

[22] L. H. Abdel‐Rahman , A. M. Abu‐Dief , R. M. El‐Khatib , and S. M. Abdel‐Fatah , “Sonochemical Synthesis, DNA Binding, Antimicrobial Evaluation and In Vitro Anticancer Activity of Three New Nano‐Sized Cu (II), Co (II) and Ni (II) Chelates Based on Tri‐Dentate NOO Imine Ligands as Precursors for Metal Oxides,” Journal of Photochemistry and Photobiology B: Biology 162 (2016): 298. 27395793 10.1016/j.jphotobiol.2016.06.052

[23] M. A. E. A. A. El‐Remaily , A. E. T. Nady , O. Elhady , et al., “A Comparative Study for the Efficiency of Pd (II) and Fe (III) Complexes as Efficient Catalysts for Synthesis of Dihydro‐7H‐5‐Thia‐Hexaaza‐s‐Indacen‐6‐One Derivatives Supported with DFT Approach,” Applied Organometallic Chemistry 38, no. 11 (2024): e7653.

[24] A. M. Abu‐Dief and L. A. E. Nassr , “Tailoring, Physicochemical Characterization, Antibacterial and DNA Binding Mode Studies of Cu (II) Schiff Bases Amino Acid Bioactive Agents Incorporating 5‐Bromo‐2‐Hydroxybenzaldehyde,” Journal of the Iranian Chemical Society 12 (2015): 943.

[25] M. A. E. A. A. El‐Remaily , O. Elhady , A. Abdou , D. Alhashmialameer , A. E. T. Nady , and A. M. Abu‐Dief , “Development of New 2‐(Benzothiazol‐2‐Ylimino)‐2,3‐Dihydro‐1H‐Imidazol‐4‐Ol Complexes as a Robust Catalyst for Synthesis of Thiazole 6‐Carbonitrile Derivatives Supported by DFT Studies,” Journal of Molecular Structure 1292 (2023): 136188.

[26] M. S. S. Adam , L. H. Abdel‐Rahman , A. M. Abu‐Dief , and N. A. Hashem , “Synthesis, Catalysis, Antimicrobial Activity, and DNA Interactions of New Cu(II)‐Schiff Base Complexes,” Inorganic and Nano‐Metal Chemistry 50, no. 30 (2020): 136–190.

[27] A. M. Abu‐Dief , E. S. Al‐Farraj , M. Abdel‐Hameed , et al., “Design and Synthesis of Tunable Schiff Base Complexes from Bis‐(2‐Oxoindolin‐3‐Ylidene) Anthracene‐9, 10‐Dione: Integrated Structural, Biological, and Molecular Modeling Insights,” Computational Biology and Chemistry 120 (2026): 108682. 40957281 10.1016/j.compbiolchem.2025.108682

[28] L. H. Abdel‐Rahman , A. M. Abu‐Dief , R. M. El‐Khatib , and S. M. A. Fatah , “Some New Nano‐Sized Fe (II), Cd (II) and Zn (II) Schiff Base Complexes as Precursor for Metal Oxides: Sonochemical Synthesis, Characterization, DNA Interaction, In Vitro Antimicrobial and Anticancer Activities,” Bioorganic Chemistry 69 (2016): 140. 27816797 10.1016/j.bioorg.2016.10.009

[29] L. H. Abdel‐Rahman , M. S. S. Adam , A. M. Abu‐Dief , et al., “Synthesis, Theoretical Investigations, Biocidal Screening, DNA Binding, In Vitro Cytotoxicity and Molecular Docking of Novel Cu (II), Pd (II) and Ag (I) Complexes of Chlorobenzylidene Schiff Base: Promising Antibiotic and Anticancer Agents,” Applied Organometallic Chemistry 32 (2018): e4527.

[30] M. A. E. A. A. El‐Remaily , O. Elhady , M. S. H. Alzubi , et al., ““Development of New Thiazole‐Guanidine Complexes as Rapid and Recoverable Catalysts for the Synthesis of 6‐Piperidin‐Dihydro‐Thia‐Hexaaza‐s‐Indacene Derivatives Supported by DFT Studies,” Applied Organometallic Chemistry 38, no. 6 (2024): e7454.

[31] M. J. Frisch , G. W. Trucks , H. B. Schlegel , et al., Gaussian 09W, Revision A.1 (Gaussian Inc, 2009).

[32] O. A. El‐Gammal , E. Abdel‐Latif , S. A. Abdel‐Latif , and H. M. El‐Sayed , “Synthesis, Characterization, Antioxidant, and Molecular Docking Studies on COVID‐19 and Breast Cancer of Novel Cr^3+^, Mn^2+^, and VO^2+^ Chelates Obtained from Novel Schiff Base Hydrazone Ligand,” Journal of Molecular Structure 1331 (2025): 141531.

[33] N. M. Alatawi , H. H. Alsharief , A. Alharbi , et al., “Simulation for the Behavior of New Fe (III) and Cr (III)‐Thiophenyl Complexes towards DNA Polymerase: Synthesis, Characterization, Eukaryotic DNA and Hartree‐Fock Computation,” Chemical Papers 76 (2022): 3919–3935.

[34] H. M. A. El‐Lateef , A. D. M. Mohamad , M. R. Shehata , and A. M. Abu‐Dief , “Targeted Synthesis of Two Iron (III) Tetradentate Dibasic Chelating Schiff Base Complexes towards Inhibition of Acidic Induced Steel Corrosion: Empirical and DFT Insights,” Applied Organometallic Chemistry 36 (2022): e6718.

[35] M. A. A. A. El‐Remaily , A. M. M. Soliman , M. E. Khalifa , et al., “Rapidly, Highly Yielded and Green Synthesis of Dihydrotetrazolo [1, 5‐a] Pyrimidine Derivatives in Aqueous Media Using Recoverable Pd (II) Thiazole Catalyst Accelerated by Ultrasonic: Computational studies,” Applied Organometallic Chemistry 36 (2022): e6320.

[36] K. Al‐Ghamdi , M. M. A. Alharas , S. A. Abdel‐Latif , et al., “Selective Novel Metal‐Coordinated Biomedical Agents Encompassing Tetradentate Salen Ligand: Structural Elucidation, DFT Calculation, Cytotoxic, and Antioxidant Activities Supported by Molecular Docking Approach,” Applied Organometallic Chemistry 39 (2025): e7991.

[37] A. M. Abu‐Dief , I. Omar , M. M. A. Alharas , et al., “Facile Synthesis, Structural Elucidation, Non‐Linear Optical Properties and Biological Evaluation of Some Novel Tetra‐Dentate Azomethine Complexes Supported by DFT Calculation and Molecular Docking Approach,” Journal of Molecular Structure 1326 (2025): 141083.

[38] A. S. Soliman , S. A. Abdel‐Latif , S. Abdel‐Khalik , S. M. Abbas , and O. M. Ahmed , “Design, Synthesis, Structural Characterization, Molecular Docking, Antibacterial, Anticancer Activities, and Density Functional Theory Calculations of Novel MnII, CoII, NiII, and CuII Complexes Based on Pyrazolone‐Sulfadiazine Azo‐Dye Ligand,” Journal of Molecular Structure 1318 (2024): 139402.

[39] A. M. Abu‐Dief , R. M. El‐Khatib , T. El‐Dabea , et al., “Design, Synthesize, Physicochemical Characterization, Nonlinear Optical Properties Structural Elucidation, Biomedical Studies, and DNA Interaction of Some New Mixed Ligand Complexes Incorporating 4,6‐Dimethylpyrimidine Derivative and Imidazole Ligand,” Applied Organometallic Chemistry 38 (2024): e7463.

[40] A. H. M. Elwahy , E. M. Eid , S. A. Abdel‐Latif , H. M. E. Hassaneen , and I. A. Abdelhamid , “Design, Synthesis, DFT, TD‐DFT/PCM Calculations, and Molecular Docking Studies on the Anti‐COVID‐19, and Anti‐SARS Activities of Some New Bis Thiazoles and Bis‐Thiadiazole,” Polycyclic Aromatic Compounds 43, no. 7 (2023): 6407–6436.

[41] O. A. Abbas , O. M. El‐Roudi , and S. A. Abdel‐Latif , “Novel 1,3‐Diphenyl‐4‐(N, N‐Dimethylimido Dicarbonimidic Diamide Azo)‐5‐Pyrazolone and Its Chelates with Manganese, Nickel, Copper, and Zinc Divalent Metal Ions as an Antibacterial Activity Supported by Molecular Docking Studies: Design, Synthesis, DFT, and TD‐DFT/PCM Calculations,” Applied Organometallic Chemistry 37 (2023): e7236.

[42] M. G. A. El‐Nasser and S. A. Abdel‐Latif , “Ligational Behavior of Bidentate Nitrogen‐Oxygen Donor 8‐Quinolinolazodye toward Ni2+ and Zn+^2^ Ions: Preparation, Spectral, Thermal, Experimental, Theoretical, and Docking Studies,” Applied Organometallic Chemistry 37 (2023): e6998.

[43] Molecular Operating Environment (MOE), version 2022.02; Chemical Computing Group ULC, 910–1010 Sherbrooke St. W., Montreal, QC H3A 2R7, Canada, 2022.

[44] A. M. Abu‐Dief , T. El‐Dabea , R. M. El‐Khatib , et al., “Fabrication, Physicochemical Characterization and Theoretical Studies of Some New Mixed Ligands Complexes Based on N‐(1H‐Benzimidazol‐2‐Yl)‐Guanidine and 1, 10‐Phenanthroline: DNA Interaction, Biological Applications and Molecular Docking Approach,” Journal of Molecular Structure 1310 (2024): 138328.

[45] A. W. Bauer , W. M. Kirby , J. C. Scherris , and M. Truck , “Antibiotic Susceptibility Testing by a Standardized Single Disk Method,” American Journal of Clinical Pathology 45 (1966): 493–496. 5325707

[46] L. H. Abdel‐Rahman , N. M. Ismail , M. Ismael , A. M. A. Dief , and E. A. H. Ahmed , “Synthesis, Characterization, DFT Calculations and Biological Studies of Mn (II), Fe (II), Co (II) and Cd (II) Complexes Based on a Tetradentate ONNO Donor Schiff Base Ligand,” Journal of Molecular Structure 1134 (2017): 851.

[47] A. Arunadevi and N. Raman , “Biological Contour, Molecular Docking and Antiproliferative Studies of DNA Targeted Histidine Based Transition Metal (II) Complexes: Invention and Its Depiction,” Applied Organometallic Chemistry 32, no. 4 (2018): e4250.

[48] F. S. Aljohani , T. El‐Dabea , R. M. El‐Khatib , et al., “Innovation, Structural Inspection for New Mixed Complexes: DNA Binding, Biomedical Applications and Molecular Docking Approaches,” Journal of Taibah University for Science 18, no. 1 (2024): 2350087.

[49] E. Ferrari , M. Asti , R. Benassi , F. Pignedoli , and M. Saladini , “Metal Binding Ability of Curcumin Derivatives: A Theoretical vs. Experimental Approach,” Dalton Transactions 42, no. 15 (2013): 5304–5313. 23403470 10.1039/c3dt33072a

[50] A. H. Bashal , K. D. Khalil , A. M. Abu‐Dief , and M. A. El‐Atawy , “Cobalt Oxide‐Chitosan Based Nanocomposites: Synthesis, Characterization and Their Potential Pharmaceutical Applications,” International Journal of Biological Macromolecules 253, no. 4 (2023): 126856. 37714231 10.1016/j.ijbiomac.2023.126856

[51] K. Nakamoto , Infrared Spectra of Inorganic and Coordination Compounds (John Wiley, 1970).

[52] K. Al‐Ghamdi , A. M. Hamed , M. A. Ibrahem , et al., “A Robust Synthesis, Physicochemical Characterization, Stability Determination and Potential Biomedical Applications of Novel Salen Complexes Supported by Theoretical Approaches,” Journal of Molecular Structure 1336 (2025): 142060.

[53] N. M. Hosny , O. A. Ibrahim , A. Belal , M. A. Hussien , and M. H. Abdel‐Rhman , “Synthesis, Characterization, DFT, Cytotoxicity Evaluation and Molecular Docking of a New Carbothioamide Ligand and Its Coordination Compounds,” Results in Chemistry 5 (2023): 100776.

[54] D. Alhashmialameer , G. G. Mohamed , Y. Al‐hawamy , et al., “Fabrication, Preparation, Physicochemical Characterization, and Theoretical Studies of Some Novel Schiff Base Ciprofloxacin Metal Complexes: DNA Interaction and Biomedical Applications,” Applied Organometallic Chemistry 38, no. 11 (2024): e7667.

[55] L. H. Abdel‐Rahman , R. M. El‐Khatib , L. A. E. Nassr , A. M. Abu‐Dief , and F. E. Lashin , “Design, Characterization, Teratogenicity Testing, Antibacterial, Antifungal and DNA Interaction of Few High Spin Fe (II) Schiff Base Amino Acid Complexes,” Spectrochimica Acta 111 (2013): 266. 23665616 10.1016/j.saa.2013.03.061

[56] I. Ali , W. A. Wani , and K. Saleem , “Empirical Formulae to Molecular Structures of Metal Complexes by Molar Conductance,” Synthesis and Reactivity in Inorganic, Metal‐Organic, and Nano‐Metal Chemistry 43, no. 9 (2013): 1162–1170.

[57] K. R. Surati and B. T. Thaker , “Synthesis, Spectral, Crystallography and Thermal Investigations of Novel Schiff Base Complexes of Manganese (III) Derived from Heterocyclic β‐Diketone with Aromatic and Aliphatic Diamine,” Spectrochimica Acta Part A 75 (2010): 235. 10.1016/j.saa.2009.10.018 19931485

[58] G. G. Mohamed , S. A. Ali , and H. F. A. El‐Halim , “Antimicrobial and Bioinformatic Modelling Studies of Isatin Mixed Ligand and Some Ternary Chelates,” ChemistrySelect 7, no. 21 (2022): e202200602.

[59] M. S. Refat , T. Altalhi , and S. B. Bakare , “New Cr(III), Mn(II), Fe(III), Co(II), Ni(II), Zn(II), Cd(II), and Hg(II) Gibberellate Complexes: Synthesis, Structure, and Inhibitory Activity Against COVID‐19 Protease,” Russian Journal of General Chemistry 91 (2021): 890–896. 34155432 10.1134/S1070363221050194 PMC8210501

[60] R. AL‐Faze , N. Alahmadi , I. O. Barnawi , et al., “Synergistic Broad‐Spectrum Bioactivity of Some Multifunctional Novel Anil Metal Chelates: Design, Synthesise, Nonlinear Optical Properties, and Biomedical Applications Supported by DFT and Molecular Docking Insights,” Journal of Molecular Structure 1339 (2025): 142390.

[61] A. M. Soliman , A. M. Abdel‐Mawgoud , W. M. A. El‐Raheem , A. M. Abu‐Dief , and A. Abdou , “New Octahedral Co (II)/Ni (II) and Square‐Pyramidal VO (II) Complexes Derived from Azo‐Schiff‐Base Ligand: Synthesis, Spectroscopic, DFT, Molecular Docking, and Biological Profiling Correlation,” Journal of Molecular Structure 1366 (2026): 146114.

[62] A. M. Abu‐Dief , A. A. Essawy , A. K. Diab , and W. S. Mohamed , “Facile Synthesis and Characterization of Novel Gd2O3‐CdO Binary Mixed Oxide Nanocomposites of Highly Hotocatalytic Activity for Wastewater Remediation under Solar Illumination,” Journal of Physics and Chemistry of Solids 148 (2021): 109666.

[63] A. M. Abu‐Dief and W. S. Mohamed , “α‐Bi2O3 Nanorods: Synthesis, Characterization and UV‐Photocatalytic Activity,” Materials Research Express 4, no. 3 (2017): 035039.

[64] W. S. Mohamed and A. M. Abu‐Dief , “Synthesis, Characterization and Photocatalysis Enhancement of Eu_2_O_3_‐ZnO Mixed Oxide Nanoparticles,” Journal of Physics and Chemistry of Solids 116 (2018): 375–385.

[65] P. Tyagi , M. Tyagi , S. Agrawal , S. Chandra , H. Ojha , and M. Pathak , “Synthesis, Characterization of 1, 2, 4‐Triazole Schiff Base Derived 3d‐Metal Complexes: Induces Cytotoxicity in HepG2, MCF‐7 Cell Line, BSA Binding Fluorescence and DFT Study,” Spectrochimica Acta Part A 171 (2017): 246. 10.1016/j.saa.2016.08.008 27541797

[66] M. A. E. A. A. El‐Remaily , O. Elhady , A. E. T. Nady , K. S. Mohamed , and A. M. A.‐ Dief , “Development of Novel Gu*a*nidine Iron (III) Complexes as a Powerful Catalyst for the Synthesis of Tetrazolo[1,5‐a] Pyrimidine by Green Protocol,” Sohag Journal of Sciences 9, no. 1 (2024): 7–15.

[67] M. Montazerozohori , S. MojahediJahromi , A. Masoudiasl , and P. McArdle , “Nano Structure Zinc (II) Schiff Base Complexes of a N3‐Tridentate Ligand as New Biological Active Agents: Spectral, Thermal Behaviors and Crystal Structure of Zinc Azide Complex,” Spectrochimica Acta Part A 5 (2015): 517. 10.1016/j.saa.2014.11.055 25528511

[68] A. W. Coats and J. P. “Redfern , “Kinetic Parameters from Thermogravimetric Data.,” Nature 201 (1964): 68e69.

[69] M. A. E. A. A. A. El‐Remaily , N. M. El‐Metwaly , T. M. Bawazeer , M. E. Khalifa , T. El‐Dabea , and A. M. Abu‐Dief , “Efficient and Recoverable Novel Pyranothiazol Pd (II), Cu (II) and Fe (III) Catalysts in Simple Synthesis of Polyfunctionalized Pyrroles: Under Mild Conditions Using Ultrasonic Irradiation,” Applied Organometallic Chemistry 35, no. 11 (2021): e6370.

[70] P. Job , “Formation and Stability of Inorganic Complexes in Solution,” Justus Liebigs Annalen der Chemie 9 (1928): 113–203.

[71] A. M. Abu‐Dief , A. M. Said , O. Elhady , et al., “Designing of Some Novel Pd(II), Ni(II) and Fe(III) Complexes: Synthesis, Structural Elucidation, Biomedical Applications, DFT and Docking Approaches against Covid‐19,” Inorganic Chemistry Communications 155 (2023): 110955.

[72] N. S. Abdel‐Kader , S. A. Abdel‐Latif , A. L. El‐Ansary , and A. G. Sayed , “Spectroscopic Studies, Density Functional Theory Calculations, Non‐Linear Optical Properties, Biological Activity of 1‐Hydroxy‐4‐((4‐(N‐ (pyrimidin‐2 Yl)sulfamoyl) Phenyl)diazenyl)‐2‐Naphthoic Acid and Its Chelates with Nickel (II), Copper (II), Zinc (II) and Palladium (II) Metal Ions,” Journal of Molecular Structure 2021 (1223): 12920.

[73] J. S. Murray and K. Sen , Molecular Electrostatic Potentials, Concepts and Applications (Elsevier, 1996).

[74] A. M. Abu‐Dief , M. A. Said , O. Elhady , et al., “Innovation of Fe(III), Ni(II), and Pd(II) Complexes Derived from Benzothiazole Imidazolidin‐4‐Ol Ligand: Geometrical Elucidation, Theoretical Calculation, and Pharmaceutical Studies,” Applied Organometallic Chemistry 37, no. 8 (2023): e7162.

[75] P. Politzer and J. S. Murray , “The Fundamental Nature and Role of the Electrostatic Potential in Atoms and Molecules,” Theoretical Chemistry Accounts 108 (2002): 134.

[76] D. Sajan , L. Joseph , N. Vijayan , and M. Karabacak , “Natural Bond Orbital Analysis, Electronic Structure, Non‐Linear Properties and Vibrational Spectral Analysis of L‐Histidinium Bromide Monohydrate: A Density Functional Theory,” Spectrochimica Acta Part A 81 (2011): 85. 10.1016/j.saa.2011.05.052 21775197

[77] D. S. Chemia and J. Zyss , Nonlinear Optical Properties of Organic Molecules and Crystals (Academic Press, 1987).

[78] D. S. Bradshaw and D. L. Andrews , “Quantum Channels in Nonlinear Optical Processes,” Journal of Nonlinear Optical Physics & Materials 18 (2009): 285.

[79] L. H. Abdel‐Rahman , A. M. Abu‐Dief , H. Moustafa , and A. A. H. Abdel‐Mawgoud , “Design and Nonlinear Optical Properties (NLO) Using DFT Approach of New Cr (III), VO (II), and Ni (II) Chelates Incorporating Tri‐Dentate Imine Ligand for DNA Interaction, Antimicrobial, Anticancer Activities and Molecular Docking Studies,” Arabian Journal of Chemistry 13, no. 1 (2020): 649–670.

[80] Y. Y. Lin , N. P. Rajesh , P. S. Raghavan , P. Ramasamy , and Y. C. Huang , “Crystal Growth of Two‐Component New Novel Organic NLO Crystals,” Materials Letters 56 (2002): 1074.

[81] L. H. Abdel‐Rahman , M. S. Adam , A. M. Abu‐Dief , H. E. Ahmed , and A. Nafady , “Non‐Linear Optical Property and Biological Assays of Therapeutic Potentials under In Vitro Conditions of Pd (II), Ag (I) and Cu (II) Complexes of 5‐Diethyl Amino‐2‐({2‐[(2‐Hydroxy‐Benzylidene)‐Amino]‐Phenylimino}‐Methyl)‐Phenol,” Molecules 25, no. 2020 (2020): 5089. 33147867 10.3390/molecules25215089 PMC7662626

[82] M. Hussain , T. Qadri , Z. Hussain , et al., “Synthesis, Antibacterial Activity and Molecular Docking Study of Vanillin Derived 1, 4‐disubstituted 1, 2, 3‐triazoles as Inhibitors of Bacterial DNA Synthesis,” Heliyon 5, no. 11 (2019): e02812. 31768438 10.1016/j.heliyon.2019.e02812 PMC6872831

[83] A. M. Abu‐Dief , R. M. El‐Khatib , T. El‐Dabea , et al., “Design, Preparation, Physicochemical Characterization, Structural Conformational, Biological Evaluation, and DNA Interaction for Some New Benzimidazole Complexes,” Applied Organometallic Chemistry 38, no. 3 (2024): e7358.

[84] V. P. Singh and A. Katiyar , “Synthesis, Characterization of Some Transition Metal(II) Complexes of Acetone p‐Amino Acetophenone Salicyloyl Hydrazone and Their anti Microbial Activity,” Biometals 21 (2008): 491–501. 18305909 10.1007/s10534-008-9136-9

[85] A. M. Abu‐Dief , O. A. Omran , M. Feizi‐Dehnayebi , et al., “Fabrication, Structural Elucidation, and DFT Calculation of Some New Hydrophilic Metal Chelates Based on NN′‐(1‐Methyl‐2‐Oxoindolin‐3‐Ylidene) Benzohydrazide Ligand: Pharmaceutical Studies and Molecular Docking Approach,” Applied Organometallic Chemistry 38, no. 9 (2024): e7593.

[86] S. K. Alharbi , S. M. Alahmadi , I. Omar , et al., “Design of Quinoline‐Derived Schiff Base Metal Complexes as Bioactive Drug Candidates: Structural Elucidation, Stability Determination, DFT, and Docking Studies with DNA‐Targeting Potential Profiles,” International Journal of Molecular Sciences 27, no. 4 (2026): 1828. 41751967 10.3390/ijms27041828 PMC12940691

[87] S. N. Pandeya , A. S. Raja , and J. P. Stables , “Synthesis of Isatinsemicarbazones as Novel Anticonvulsants‐Role of Hydrogen Bonding,” Journal of Pharmacy & Pharmaceutical Sciences 5 (2002): 266–271. 12553895

[88] B. Tweedy , “Plant Extracts with Metal Ions as Potential Antimicrobial Agents,” Phytopathology 55 (1964): 910–914.

[89] N. A. Illán‐Cabeza , A. R. García‐García , M. N. Moreno‐Carretero , J. M. Martínez‐Martos , and M. J. Ramírez‐Expósito , “Synthesis, Characterization and Antiproliferative Behavior of Tricarbonyl Complexes of Rhenium(I) with Some 6‐Amino‐5‐Nitrosouracil Derivatives. Crystal Structure of Fac‐[ReCl(CO)3(DANU‐N5O4)] (DANU = 6‐Amino‐1,3‐dimethyl5‐Nitrosouracil),” Journal of Inorganic Biochemistry 99 (2005): 1637–1645. 15964633 10.1016/j.jinorgbio.2005.05.003

[90] L. M. Gaetke and C. K. Chow , “Copper Toxicity, Oxidative Stress, and Antioxidant Nutrients,” Toxicology 189 (2003): 147–163. 12821289 10.1016/s0300-483x(03)00159-8

[91] A. Choudhary , R. Sharma , M. Nagar , M. Mohsin , and H. S. Meena , “Synthesis, Characterization and Antioxidant Activity of Some Transition Metal Complexes with Terpenoid Derivatives,” Chemical Society 56 (2011): 911–917. 10.3109/14756366.2010.518966 20939764

[92] N. Farhan , R. S. Abo‐Rehab , M. R. Shehata , et al., “Engineered for Impact: Multifunctional Co (II), Ni (II), Cu (II), and Cd (II) Complexes of 2‐Aminobenzothiazole with Potent Antitumor, Antibacterial, and Antioxidant Actions Supported by Theoretical Approaches,” Journal of Molecular Structure 1351 (2026): 144295.

