# Supplementary material for: Ultrasound‐Assisted Synthesis and Comprehensive Characterization of Nanosized VO(II), Fe(III), and Ru(III) Complexes: From Density Functional Theory/Nonlinear Optical Properties to Pharmaceutical Applications and Docking Insights
Source: ChemistryOpen. 2026 May 22;15(6):e70229. doi: 10.1002/open.70229 (PMC13240355; doi:10.1002/open.70229)
Supplement: Supplementary file 1 — Supplementary Material [file OPEN-15-e70229-s001.pdf]

2.2 | Reagent and Instrumentation

All starting materials and solvents were used as received from Sigma Aldrich or Alfa Aesar. Thin-layer chromatography was conducted using TLC silica gel 60 F_{254} (Merck Co.), visualized with ultraviolet light. All melting points were recorded on Melt-Temp II melting point apparatus. IR spectra were measured as KBr pellets on a Shimadzu DR-8001 spectrometer. ^{1}H, ^{13}C NMR spectra were recorded on a Bruker DRX 400 MHz using TMS as an internal reference and DMSO as a solvent. All compounds were checked for their purity on TLC plates. By using model T+80 of PG spectrophotometer in DMF as a solvent were detected UV-vis spectra of the ligand and it's complexes at 298 K at the University of Sohag, Faculty of Science in the Department of Chemistry. The elemental [C, H & N] analyses were done at the Central Laboratory, University of Zagazig by [Perkin-Elmer 2408] analyzer. By using model 60 H of Shimaduz analyzer for thermo gravimetric analysis and determine the parameters of thermo-kinetic activation of synthesized complexes in air and at a heating rate of 10°C min^{-1} in the Department of Chemistry, Faculty of Science, Thermal Analysis Unit, the University of Cairo, Egypt. For estimation the molar conductance of the complexes at 298K. By utilizing model 4320 of conductivity meter [JENWAY] in DMF as a solvent. By using Elico digital [model LI-127] pH meter comes with a [CL-51B] combined electrode for pH evaluation and before evaluations might be calibrate vs standard buffers of Britton global buffers. Electronic spectra were estimated in DMF at 298 K using a Jasco P-530 UV– vis spectrophotometer.

2.8 | Spectrophotometric studies

The UV-Vis spectroscopic analysis conducted over a broad range of wavelengths, from 200 nm to 800 nm. This wavelength interval is ideal because it encompasses the typical absorption peaks of metal complexes, which often occur in the visible and ultraviolet regions. These peaks are associated with electronic transitions in the metal centers and can provide insight into the nature of the metal-ligand interactions, the geometry of the complex, and the presence of any ligand-to-metal charge transfer (LMCT) or metal-to-ligand charge transfer (MLCT) processes.

2.11.3 | Antioxidant activity

The evaluation of the antioxidant capabilities of the molecules in question was performed using the DPPH free radical scavenging method, as outlined in prior research [42]. This assay, known for its widespread application and simplicity, fundamentally relies on the process of antioxidants diminishing the stable DPPH radical. In the experimental setup, a 2.0 mL sample of the chelates under examination is blended with 6.0 mL of the DPPH reagent. The blend is then placed in an environment devoid of light at a consistent temperature of 25 °C, and is granted a 30-minute duration for the chemical interaction to unfold. After the incubation phase, the absorbance of each solution was measured at 519 nm using a spectrophotometer, with reference to control and reference samples. It is noteworthy that the control group did not contain the test chelates. The IC_{50} value for DPPH was computed by applying the given equation:

$$\text{Scavenging activity (\%)} = {A_\circ - A_t}/{A_\circ} \times 100$$

Here, A_0 represents the absorbance at the initial time point (t=0) for the control (standard) sample, while A_t denotes the absorbance at the conclusion of the 30-minute incubation for the solution containing the antioxidants. To ensure accuracy and reliability, the assay involved a series of dilutions corresponding to varying doses and was replicated three times for each experimental condition [43].

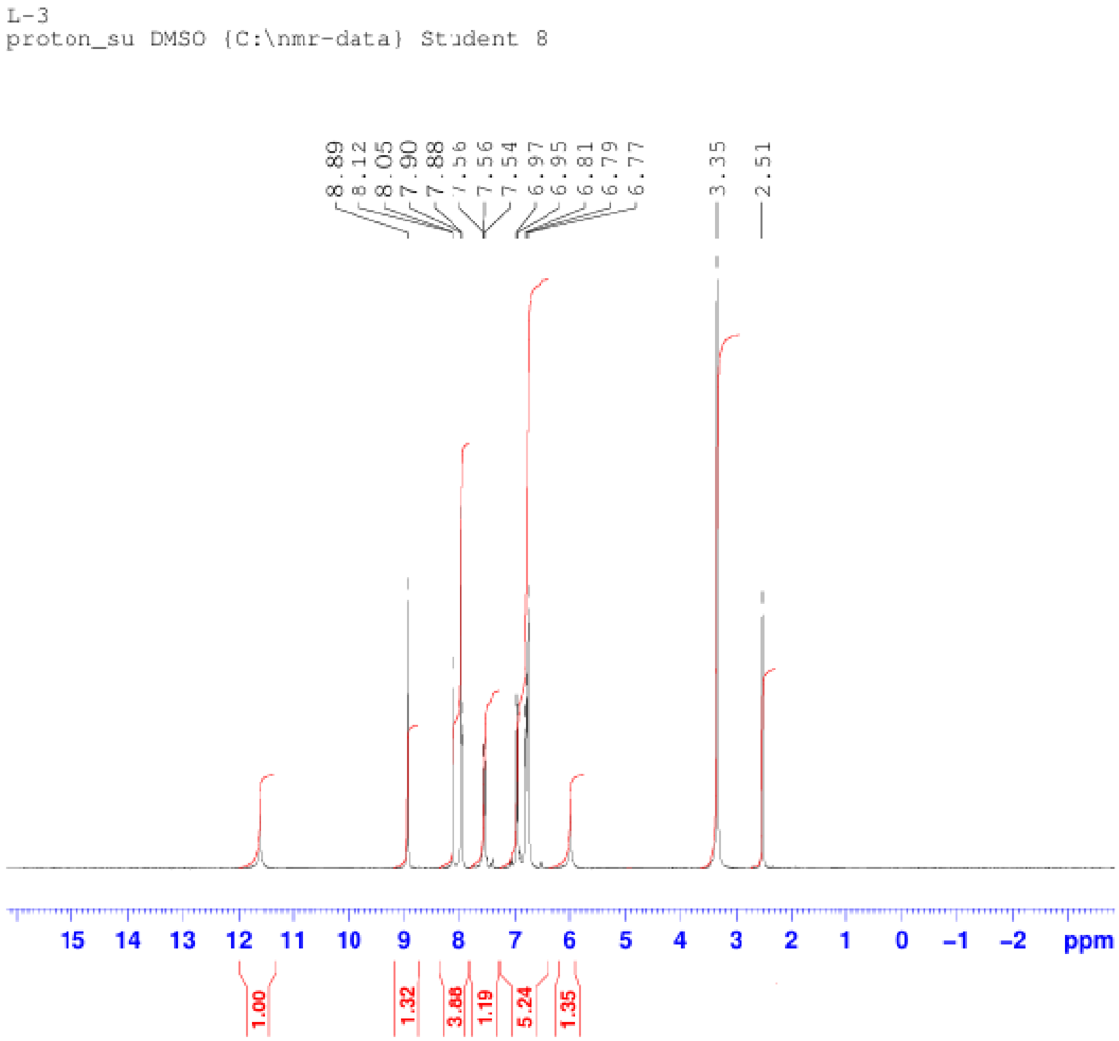

^{1}H-NMR (∂, ppm), in DMSO-d_6 (Fig 1S) of CHBPI ligand

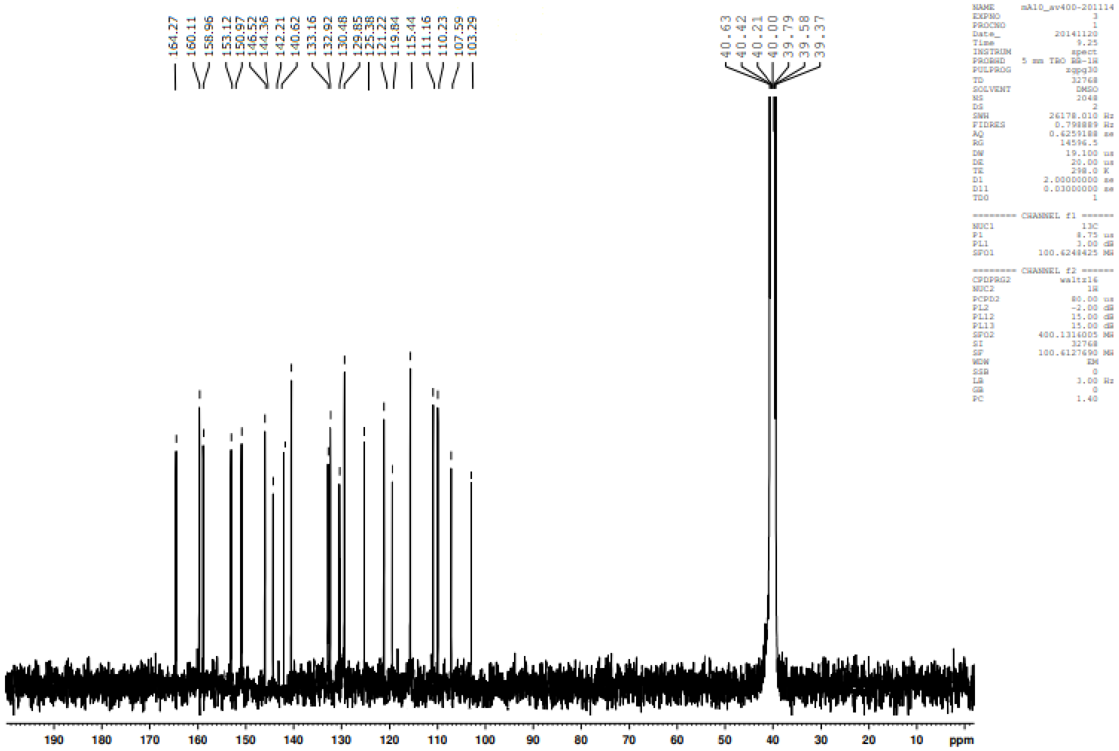

^{13}C-NMR (∂, ppm), in DMSO-d_6 (Fig 2S) of CHBPI ligand

(Table 1S): Molecular electronic spectra, λ_{max} (nm), ε_{max} (dm^3 mol^{-1} mm^{-1}) of the synthesized CHBPI ligand and its complexes in DMF at 298 K against DMF as a blank.

CHBPI ligand and its complexes	λ_{max} (nm)	ε_{max} (dm^3 mol^{-1} cm^{-1})	Assignment
**CHBPI**	422<br>368<br>295<br>232	1568<br>1444<br>1309<br>1166	Intra ligand band<br>n →π*<br>π→π*<br>π→π*
**CHBPIV**	448<br>386<br>300<br>257	1895<br>1608<br>1240<br>1051	d-d band<br>LMCT band<br>n→π*<br>π→π*
**CHBPIFe**	522<br>459<br>372<br>332<br>318<br>291	1255<br>1841<br>1424<br>1175<br>1017<br>1389	d-d band<br>LMCT band<br>LMCT band<br>n→π*<br>n→π*<br>π→π*
**CHBPIRu**	553<br>473<br>399<br>319<br>291	1543<br>1925<br>1126<br>754<br>714	d-d band<br>LMCT band<br>LMCT band<br>n→π*<br>π→π*

(Table 2S): Selected geometric bond lengths, bond angles, and dihedral angles of the optimized CHBPI ligand and its Fe, Ru, and VO chelates using B3LYP/6-311G(d,p) and B3LYP/6-311G(d,p)-LANL2DZ level

Compound	Bond lengths (Å)		Bond angles		Dihedral angles	
Ligand (CHBPI)	C18-O20	1.2024	O20-C18-C14	128.405	O20-C18-C14-N11	-1.468
	C18-C14	1.5414	C18-C14-N11	118.815	C18-C14-N11-C6	175.125
	C14-N11	1.2709	C14-N11-C6	124.461	C14-N11-C6-C5	118.317
	C6-N11	1.3962	N11-C6-C5	118.945	N11-C6-C5-N10	-3.985
	C6-C5	1.4152	C6-C5-N10	117.257	C6-C5-N10-C13	-148.197
	C5-N10	1.3983	C5-N10-C13	122.316	C5-N10-C13-C29	-176.524
	N10-C13	1.2869	N10-C13-C29	121.937	N10-C13-C29-C31	-1.041
	C13-C29	1.4490	C13-C29-C31	121.389	C13-C29-C31-O39	-0.237
	C31-O39	1.3343	O39-C31-C39	122.793	O39-C31-C39-C13	-0.237
Fe-CHBPI	C5-C6	1.4199	C5-C6-N11	114.093	C5-C6-N11-C14	-169.027
	C5-N10	1.4098	C5-N10-Fe41	113.122	C5-N10-Fe41-O30	8.015
	C6-N11	1.3920	C6-N11-C14	134.081	C6-N11-C14-C16	-173.549
	N11-C14	1.2973	N11-C14-C16	111.980	N11-C14-C16-O30	-2.870
	C14-C16	1.5169	C14-C16-O30	123.876	C14-C16-O30-Fe41	-6.168
	C16-O30	1.2336	C16-O30-Fe41	106.410	C16-O30-Fe41-O40	-177.685
	O30-Fe41	2.0902	O30-Fe41-O40	98.849	O30-Fe41-O40-C33	153.502
	Fe41-O40	1.8349	Fe41-O40-C33	125.198	Fe41-O40-C33-C15	18.397
	O40-C33	1.3084	O40-C33-C15	123.186	O40-C33-C15-C13	2.949
	C33-C15	1.4356	C33-C15-C13	122.526	C33-C15-C13-N10	-5.016
	C15-C13	1.4189	C15-C13-N10	125.211	C15-C13-N10-Fe41	-13.231
	C13-N10	1.3094	C13-N10-Fe41	122.194	C13-N10-Fe41-N11	-161.580
	N10-Fe41	1.9116	N10-Fe41-N11	83.742	N10-Fe41-N11-C6	-7.989
Ru-CHBPI	C5-C6	1.4115	C5-C6-N11	115.421	C5-C6-N11-C13	-161.492
	C5-N10	1.4320	C5-N10-Ru40	117.441	C5-N10-Ru40-O38	11.508
	C6-N11	1.3875	C6-N11-C13	125.077	C6-N11-C13-C16	-174.866
	N11-C13	1.3163	N11-C13-C16	111.403	N11-C13-C16-O38	-5.256
	C13-C16	1.4974	C13-C16-O38	121.173	C13-C16-O38-Ru40	-2.102
	C16-O38	1.2430	C16-O38-Ru40	116.574	C16-O38-Ru40-O37	-169.678
	O38-Ru40	2.3201	O38-Ru40-O37	129.354	O38-Ru40-O37-C31	178.841
	Ru40-O37	2.0332	Ru40-O37-C31	133.346	Ru40-O37-C31-C28	-4.958
	O37-C31	1.2999	O37-C31-C28	123.118	O37-C31-C28-C14	0.123
	C31-C28	1.4203	C31-C28-C14	122.483	C31-C28-C14-N10	-0.518

	C28-C14	1.4102	C28-C14-N10	130.739	C28-C14-N10-Ru40	4.797
	C14-N10	1.3110	C14-N10-Ru40	123.544	C14-N10-Ru40-N11	176.136
	C14-N10	1.3110	C14-N10-C5	118.625	C14-N10-C5-C6	-170.466
**VO-CHBPI**	C5-C6	1.4222	C5-C6-N12	113.596	C5-C6-N12-C14	158.934
	C5-N11	1.4162	C5-N11-V41	112.510	C5-N11-V41-O28	-71.867
	C6-N12	1.3934	C6-N12-C14	132.409	C6-N12-C14-C16	178.017
	N12-C14	1.2995	N12-C14-C16	111.662	N12-C14-C16-O28	2.416
	C14-C16	1.5153	C14-C16-O28	122.960	C14-C16-O28-V41	-1.846
	C16-O28	1.2364	C16-O28-V41	111.548	C16-O28-V41-O40	138.626
	O28-V41	2.1204	O28-V41-O40	93.766	O28-V41-O40-C35	-171.044
	V41-O40	1.8796	V41-O40-C35	131.389	V41-O40-C35-C29	22.252
	O40-C35	1.3120	O40-C35-C29	122.299	O40-C35-C29-C13	-0.104
	C35-C29	1.4323	C35-C29-C13	122.217	C35-C29-C13-N11	-7.318
	C29-C13	1.4199	C29-C13-N11	125.561	C29-C13-N11-V41	-5.065
	C13-N11	1.3159	C13-N11-V41	125.169	C13-N11-V41-N12	156.845
	C13-N11	1.3159	C13-N11-C5	122.265	C13-N11-C5-C6	-159.85
	N11-C5	1.4162	N11-C5-C6	115.57	N11-C5-C6-N12	-0.272

(Table 3S): Calculated natural population, natural charge, and natural electronic configuration of the metal in the studied Fe, Ru, and VO chelates using B3LYP/6-311G(d,p)-LANL2DZ level

Complex	Natural charge	Natural population				Natural electronic configuration
		Core	Valence	Rydberg	Total	
CHBPIFe	0.4299	17.9902	7.5470	0.0330	25.5701	[core]$4s^{0.25}3d^{6.86}4p^{0.01}4d^{0.02}5p^{0.44}$
CHBPIRu	0.5518	35.9877	7.4353	0.0252	43.4482	[core]5s0.21$4d^{6.86}5p^{0.13}5d^{0.02}6p^{0.25}$
CHBPIV	0.7504	17.9716	4.2299	0.0481	22.2497	[core]$4s^{0.22}3d^{3.71}4p^{0.20}4d^{0.04}5p^{0.11}$

(Table 4S): The Docking interaction data calculations of the CHBPI ligand and its complexes with the active site of the receptor of Breast cancer (PDB ID: 3HB5), *Candida albicans* (PDB ID: 3QLW), and *Micrococcus luteus* (PDB ID: 3IF5)

System	Binding score (kcal/mol)	Receptor	Interaction	Distance(Å)	E (kcal/mol)
**Breast cancer (PDB ID: 3HB5**					
**CHBPI-3HB5**<br>**6-ring**	-7.39	N VAL 188	pi-H	4.04	-0.7
**Fe-CHBPI-3HB5**<br>**Cl 41**<br>**O 44**<br>**6-ring**	-5.19	O LEU 95<br>OG SER 142<br>CE1 PHE 226	H-donor<br>H-donor<br>pi-H	3.44<br>2.93<br>4.53	-0.5<br>-2.4<br>-0.6
**Ru-CHBPI-3HB5**<br>**-**	-4.22		-	-	-
**VO-CHBPI-3HB5**<br>**O 45**	-6.24	O GLY 186	H-donor	2.91	-0.9
**Cisplatin-3HB5**					
**Cl 10**	-3.41	N GLY 92	H-acceptor	3.39	-0.8
***Candida albicans* (PDB ID: 3QLW)**					
**CHBPI-3QLW**<br>**O 38**<br>**C 11**	-6.44	OE1 GLU 32<br>6-ring PHE 36	H-donor<br>H-pi	2.91<br>3.83	-2.8<br>-0.8
**Fe-CHBPI-3QLW**<br>**O 45**	-6.51	6-ring PHE 36	H-pi	4.72	-1.2
**Ru-CHBPI-3QLW**					
**C 3**<br>**Cl 43**<br>**6-ring**	-5.99	SD MET 25<br>NZ LYS 37<br>6-ring PHE 66	H-donor<br>H-acceptor<br>pi-pi	4.09<br>3.44<br>3.61	-1.0<br>-1.0<br>-0.0
**VO-CHBPI-3QLW**					
**-**	-5.66	-	-	-	-
**Fluconazole-3QLW**					
N 31	-5.74	N ARG 79	H-acceptor	3.03	-0.9
N 34		NH2 ARG 56	H-acceptor	2.78	-4.1
6-ring		CB LYS 57	pi-H	4.18	-0.8
***Micrococcus luteus* (PDB ID: 3IF5)**					
**CHBPI-3IF5**<br>**-**	-6.02	-	-	-	-
**Fe-CHBPI-3IF5**					
**6-ring**	-5.52	N LEU 312	pi-H	4.16	-0.7
**Ru-CHBPI-3IF5**<br>**O 31**<br>**O 46**	-5.47	OD2 ASP 419<br>OD2 ASP 419	H-donor<br>H-donor	2.83<br>2.94	-13.6<br>-5.8
**VO-CHBPI-3IF5**					
**N 10**<br>**V 44**<br>**N 10**<br>**N 10**<br>**C 15**		OD1 ASP 419<br>OD2 ASP 419<br>OD1 ASP 419<br>OD2 ASP 419	H-donor<br>metal<br>ionic<br>ionic<br>ionic	3.13<br>1.92<br>3.13<br>2.91<br>3.72	-3.3<br>-2.7<br>-3.7<br>-5.1<br>-1.2

**C 15**<br>**C 24**<br>**C 24**<br>**N 29**<br>**O 31**<br>**O 45**	-7.02	NH1 ARG 422<br>NH2 ARG 422<br>NH1 ARG 422<br>NH2 ARG 422<br>OD2 ASP 419<br>OD2 ASP 419<br>OD2 ASP 419	ionic<br>ionic<br>ionic<br>ionic<br>ionic<br>ionic	3.78<br>3.60<br>3.45<br>3.66<br>2.59<br>3.56	-1.0<br>-1.5<br>-2.1<br>-1.3<br>-7.9<br>-1.7
**Ofloxacin-3IF5**					
**-**	-5.66	-	-	-	-

(**Table 5S):** Antibacterial activity inhibition zone of the prepared compounds against the selected strains of bacteria

Compounds	**Inhibition zone (mm)**					
	Escherichia coli (-ve)		*Serratia Marcescence (-ve)*		***Micrococcus Luteus (+ve)***	
**Conc. (µg/ml)**	15	25	15	25	15	**25**
**CHBPI**	6.25±0.04	9.80±0.05	9.15±0.10	15.75±0.12	11.85±0.15	17.20±0.06
**CHBPIV**	14.90±0.17	25.30±0.08	17.25±0.11	31.80±0.08	21.85±0.16	41.35±0.17
**CHBPIFe**	12.10±0.13	22.55±0.04	15.30±0.17	30.20±0.07	20.25±0.09	39.85±0.14
**CHBPIRu**	16.25±0.09	26.75±0.06	18.75±0.13	33.65±0.14	23.45±0.10	43.65±0.09
Ofloxacin	16.85±0.10	28.35±0.19	20.40±0.17	35.70±0.09	25.85±0.13	45.40±0.15

(**Table 6S):** Antifungal activity inhibition zone of the prepared compounds against the selected strains of fungi

Compounds	**Inhibition zone (mm)**					
	Fusarium oxysporum		*Candida albicans*		**Aspergillus flavus**	
**Conc. (µg/ml)**	15	25	15	25	15	**25**
**CHBPI**	7.85±0.07	12.45±0.16	10.30±0.05	14.50±0.08	6.50±0.08	10.70±0.09
**CHBPIV**	18.75±0.16	30.40±0.07	23.50±0.14	37.65±0.17	13.10±0.05	24.45±0.10
**CHBPIFe**	16.45±0.17	28.95±0.10	21.40±0.07	35.90±0.06	11.75±0.04	23.65±0.14
**CHBPIRu**	20.20±0.10	31.80±0.16	24.10±0.07	39.80±0.10	4.20±0.071	25.40±0.11
Fluconazole	21.85±0.13	33.65±0.15	25.30±0.02	41.15±0.14	15.60±0.17	26.10±0.16

(Table 7S): Cytotoxic activity (IC_{50}) of the prepared compounds against Colon carcinoma cells, (HCT-116 cell line) and hepatic cellular carcinoma cells, (HepG-2).and breast carcinoma cells (MCF-7) for CHBPI ligand and its metal chelates

Compounds	IC_{50}(µg/µl)		
	HCT-116	MCF-7	HepG-2
CHBPI	19.45±0.13	14.75±0.17	16.55±0.10
CHBPIV	7.95±0.08	5.12±0.11	6.87±0.15
CHBPIFe	9.25±0.10	6.72±0.04	8.10±0.05
CHBPIRu	6.95±0.08	3.75±0.14	5.07±0.17
Cisplatin	8.45±0.12	10.45±0.14	6.35±0.08

(Table 8S): Antioxidant activity of the investigated compounds

Complex	IC50
CHBPI	58.90±0.07
CHBPIV	19.75±0.04
CHBPIFe	23.68±0.17
CHBPIRu	14.35±0.08
Ascorbic acid	54.50±0.13
